# CRISPR/Cas9-Mediated Genome Editing of Herpesviruses Limits Productive and Latent Infections

**DOI:** 10.1371/journal.ppat.1005701

**Published:** 2016-06-30

**Authors:** Ferdy R. van Diemen, Elisabeth M. Kruse, Marjolein J. G. Hooykaas, Carlijn E. Bruggeling, Anita C. Schürch, Petra M. van Ham, Saskia M. Imhof, Monique Nijhuis, Emmanuel J. H. J. Wiertz, Robert Jan Lebbink

**Affiliations:** 1 Medical Microbiology, University Medical Center Utrecht, Utrecht, The Netherlands; 2 Department of Ophthalmology, University Medical Center Utrecht, Utrecht, The Netherlands; Oregon Health and Science University, UNITED STATES

## Abstract

Herpesviruses infect the majority of the human population and can cause significant morbidity and mortality. Herpes simplex virus (HSV) type 1 causes cold sores and herpes simplex keratitis, whereas HSV-2 is responsible for genital herpes. Human cytomegalovirus (HCMV) is the most common viral cause of congenital defects and is responsible for serious disease in immuno-compromised individuals. Epstein-Barr virus (EBV) is associated with infectious mononucleosis and a broad range of malignancies, including Burkitt’s lymphoma, nasopharyngeal carcinoma, Hodgkin’s disease, and post-transplant lymphomas. Herpesviruses persist in their host for life by establishing a latent infection that is interrupted by periodic reactivation events during which replication occurs. Current antiviral drug treatments target the clinical manifestations of this productive stage, but they are ineffective at eliminating these viruses from the infected host. Here, we set out to combat both productive and latent herpesvirus infections by exploiting the CRISPR/Cas9 system to target viral genetic elements important for virus fitness. We show effective abrogation of HCMV and HSV-1 replication by targeting gRNAs to essential viral genes. Simultaneous targeting of HSV-1 with multiple gRNAs completely abolished the production of infectious particles from human cells. Using the same approach, EBV can be almost completely cleared from latently infected EBV-transformed human tumor cells. Our studies indicate that the CRISPR/Cas9 system can be effectively targeted to herpesvirus genomes as a potent prophylactic and therapeutic anti-viral strategy that may be used to impair viral replication and clear latent virus infection.

## Author Summary

Herpesviruses are large DNA viruses that are carried by almost 100% of the adult human population. Herpesviruses include several important human pathogens, such as herpes simplex viruses (HSV) type 1 and 2 (causing cold sores and genital herpes, respectively), human cytomegalovirus (HCMV; the most common viral cause of congenital defects, and responsible for serious disease in immuno-compromised individuals), and Epstein-Barr virus (EBV; associated with infectious mononucleosis and a wide range of malignancies). Current antiviral drug treatments are not effective in clearing herpesviruses from infected individuals. Therefore, there is a need for alternative strategies to combat these pathogenic viruses and prevent or cure herpesvirus-associated diseases. Here, we have assessed whether a direct attack of herpesvirus genomes within virus-infected cells can inactivate these viruses. For this, we have made use of the recently developed CRISPR/Cas9 genome-engineering system to target and alter specific regions within the genome of these viruses. By targeting sites in the genomes of three different herpesviruses (HSV-1, HCMV, and EBV), we show complete inhibition of viral replication and in some cases even eradication of the viral genomes from infected cells. The findings presented in this study open new avenues for the development of therapeutic strategies to combat pathogenic human herpesviruses using novel genome-engineering technologies.

## Introduction

Herpesviruses are large DNA viruses that cause widespread, lifelong infections; most adults carry multiple herpesviruses [1]. The herpesvirus family is divided into three subfamilies, the *Alpha-*, *Beta-* and *Gammaherpesvirinae*. The subfamily of *Alphaherpesvirinae* includes the herpes simplex virus type 1 and type 2 (HSV-1 and 2) and varicella zoster virus (VZV). HSV-1 causes cold sores and herpes simplex keratitis, a common cause of corneal blindness [2, 3]. HSV-2 is responsible for genital herpes. Primary infection with VZV results in chickenpox; reactivation may lead to herpes zoster or shingles [4]. The subfamily of *Betaherpesvirinae* includes the human cytomegalovirus (HCMV), which gives rise to serious complications in immuno-compromised individuals [5, 6]. Additionally, HCMV is the most common viral cause of congenital defects. The *Gammaherpesvirinae* include Epstein-Barr virus (EBV) and Kaposi's sarcoma-associated herpesvirus (KSHV). EBV induces infectious mononucleosis and is strongly associated with multiple malignancies, including nasopharyngeal carcinoma, Burkitt’s lymphoma, Hodgkin’s lymphoma, gastric carcinoma, and post-transplant lymphoproliferative disorders (PTLD) [7]. KSHV is a human tumor virus that is associated with Kaposi's sarcoma and two lymphoproliferative disorders occurring in AIDS patients: primary effusion lymphoma and multicentric Castleman disease [8].

Current treatment options to restrict the clinical manifestations of productive herpesvirus infections are limited and all approved antiviral agents target the viral DNA polymerase [9, 10] during the productive (lytic) phase of infection. Herpesviruses, however, are characterized by their ability to establish a quiescent, latent state [1]. During latency, herpesviruses express only few viral gene products allowing them to persist in the host without being effectively cleared by our immune system. During this stage, herpesviruses are not actively replicating their viral genomes by viral DNA polymerases, rendering antiviral treatments targeting these polymerases ineffective. Occasionally, herpesviruses reactivate, thereby producing new virus progeny; depending on the herpesvirus in question, reactivation may cause serious disease [1]. Examples are herpes simplex keratitis, genital herpes and herpes zoster, caused by HSV-1, HSV-2 and VZV, respectively. For EBV, pathology is mainly associated with latent infection which is associated with the occurrence of various tumors of lymphoid and epithelial origin [11]. There is a need to clear the latent infection, as this would allow the removal of herpesvirus pathogens from the human host; this would prevent future reactivation events and herpesvirus-associated diseases.

Archaea and bacteria have evolved an adaptive immune system comprised by CRISPR/Cas (clustered regularly interspaced short palindromic repeats (CRISPR)—CRISPR–associated (Cas) systems) that use short RNA molecules to induce degradation of foreign nucleic acids of viruses and other genetic elements [12–15]. Recently, this CRISPR/Cas9 system has been engineered to induce robust RNA-guided genome modifications in human cells [16]. By co-expressing a bacterial Cas9 nuclease with a guideRNA (gRNA), one can direct Cas9 to almost any site in the genome and induce cleavage of double stranded DNA (dsDNA) at the target site [16]. This cleaved DNA is subsequently ‘repaired’ by mammalian DNA repair mechanisms that are inherently error-prone, thereby inducing insertions and deletions (‘indels’) and mutations at the target site. Applications of the CRISPR/Cas9 system are currently revolutionizing the field of molecular biology. Researchers are now able to generate full knockouts for any gene in any cell-type [17, 18], induce specific editing/mutagenesis of loci [15, 17, 19], edit multiple alleles simultaneously [20, 21], in addition to many other applications [12, 15, 22]. The CRISPR/Cas9 system has also been engineered to selectively modify dsDNA viruses [23–30], positive-sense single stranded RNA viruses, such as Hepatitis C [31] and integrated HIV proviral DNA in human cells [32–36]. Therefore, CRISPR/Cas9 targeting of herpesviral genomes may provide a powerful strategy to combat these viruses.

In this study, we set out to explore whether the CRISPR/Cas9 system can be rewired to limit herpesvirus infection during the latent and productive stage of the viral life cycle. For this, we usurped the CRISPR/Cas9 system to induce efficient editing of the genomes of three prototypic members of the human herpesvirus family. By targeting gRNAs to essential viral genes, we show effective abrogation of HCMV and HSV-1 replication. Simultaneous targeting of HSV-1 with two gRNAs completely impaired the production of new infectious particles from human cells. Using the same approach, EBV can be efficiently cleared from latently infected EBV-transformed human tumor cells. On the contrary, CRISPR/Cas9 appears inefficient at targeting quiescent HSV-1 genomes whereas replication post virus reactivation can be efficiently abrogated. The data presented in this study indicate the potential of the CRISPR/Cas9 system as a new therapeutic strategy to combat pathogenic human herpesviruses.

## Results

### Direct editing of EBV genomes using the CRISPR/Cas9 system

**Fig 1:**
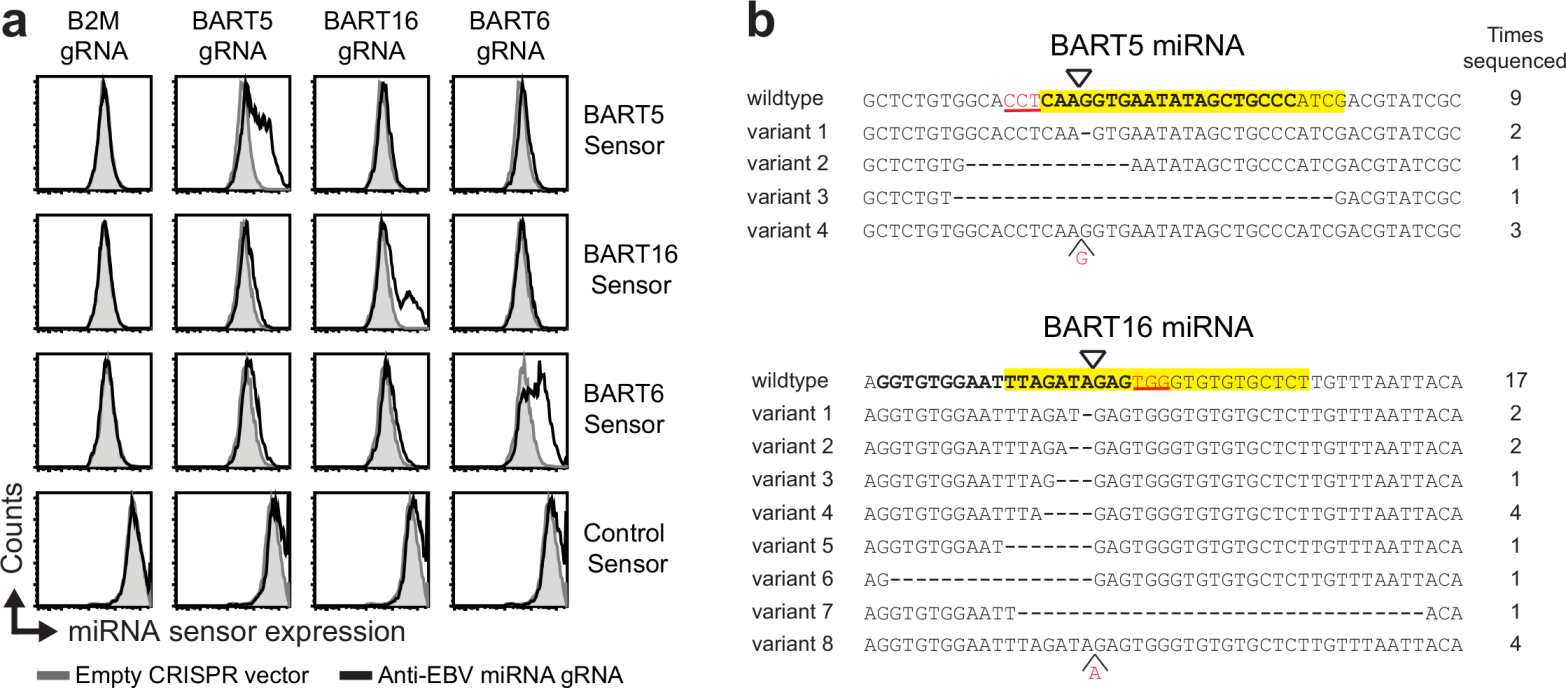
Editing of the EBV genome in latently infected tumor cells using CRISPR/Cas9. **a)** Latently infected gastric carcinoma SNU719 cells were transduced with lentiviral CRISPR/Cas vectors targeting the indicated EBV miRNA genes. The lines were subsequently selected with puromycin for 2 days and allowed to recover for 12 days. The activity of the targeted miRNAs was subsequently monitored by introduction of the indicated miRNA sensor vectors and assessment of the mCherry reporter expression after 4 days. Increased sensor expression indicates a loss of EBV miRNAs. The activities for the EBV miRNAs in the absence of gRNAs is presented in S1 Fig. **b)** Sequencing of CRISPR-targeted EBV genomes indicates editing at the target sites. The EBV genomic locus of BART5 and BART16 were amplified by PCR, cloned in a DNA cloning vector, and subjected to Sanger DNA sequencing. The miRNA sequence is presented in yellow, the gRNA-target sites are displayed in bold, the PAM sequence as red, underlined text, and the cleavage site as a triangle. The number of times each variant has been sequenced is indicated.

We set out to explore whether the CRISPR/Cas9 system could be targeted to the genome of human herpesviruses to directly limit virus replication in human cells. We generated a lentiviral CRISPR/Cas9 vector and asked whether human herpesviruses within infected cells can be genetically modified using this system. We initially focused our efforts on EBV, whose dsDNA genome resides in the nucleus of infected cells [7]. EBV can establish latency in B-lymphocytes, where the EBV genome circularizes and resides as an episome in the nucleus. During latency, the virus does not produce virus progeny [7] and expresses a limited number of viral proteins, in addition to non-coding RNAs, including a large set of miRNAs. We assessed whether these viral miRNA genes were amendable to CRISPR/Cas9 editing (see S1C Fig for a graphical representation of the experimental setup). The latently infected gastric carcinoma cell line SNU-719 was transduced to express CRISPR/Cas9 gRNAs targeting the viral miRNAs BART5, BART6, or BART16. The relative miRNA activity of these specific miRNAs was assessed using miRNA sensor constructs in which a single miRNA target site was cloned downstream of the fluorescent mCherry reporter (S1B Fig). Introduction of the miRNA sensors in SNU-719 cells resulted in downregulation of mCherry expression (S1B Fig), confirming functional expression of the miRNAs in these cells. Expression of the sensors was partially restored upon treatment of the cells with gRNAs targeting the corresponding miRNAs (Fig 1A), indicating site-specific editing of the EBV genome. Indeed, sequencing of the genomic miRNA loci showed editing of the targeted regions (Fig 1B). Hence, the CRISPR/Cas9 system allows for direct editing of the genome of latent EBV in EBV-positive tumor cells.

### CRISPR/Cas9-mediated targeting of essential EBV genetic elements clears EBV from latently infected lymphoma cells

**Fig 2:**
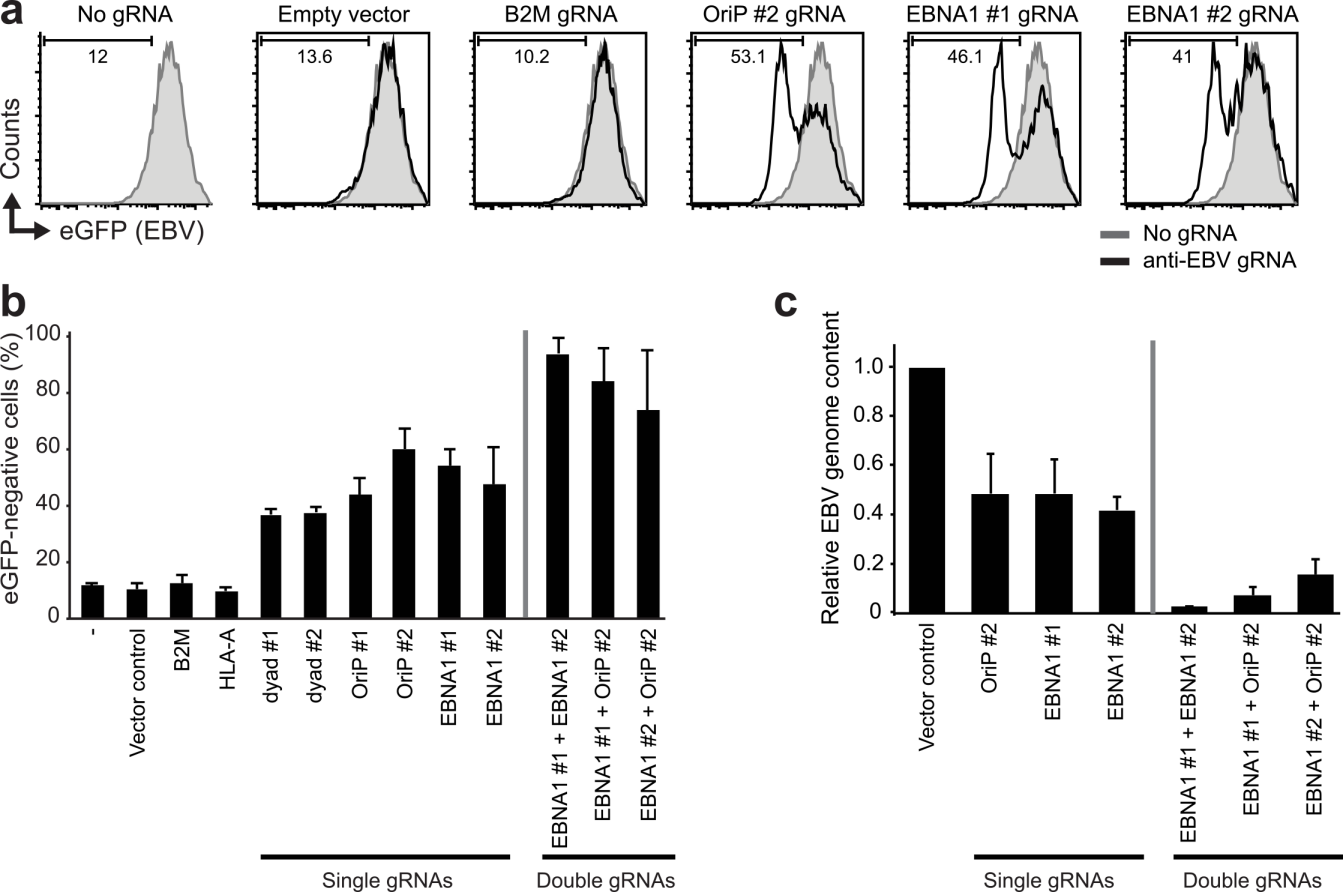
CRISPR/Cas9-mediated clearance of EBV genomes from latently infected Burkitt’s lymphoma cells. **a)** Anti-EBV gRNAs induce a potent loss of EBV genomes from latently infected cells. Burkitt’s lymphoma Akata-Bx1 cells latently infected with eGFP-EBV (endogenously driving eGFP expression) were transduced with anti-EBV gRNAs targeting EBNA1, OriP, or control genes and selected with puromycin for 2 days. Subsequently, the cells were monitored for the presence of EBV-eGFP by flow cytometry 21 days post transduction. The percentage of eGFP negative cells as measure for EBV-eGFP loss is indicated. **b)** Combinatorial anti-EBV gRNA treatment of Akata-Bx1 cells causes increased loss of EBV genomes from latently infected cells. Similar experimental set-up as in a), but with a larger set of anti-EBV gRNAs and combinations thereof introduced through sequential application of two separate CRISPR vectors. The percentage of EBV-eGFP negative cells is presented. **c)** Samples from b) were subjected to qPCR to quantify the relative EBV genome content in the indicated gRNA-expressing Akata-Bx1 cells. Since the amplified region in the qPCR lies outside the genomic region that is targeted by the gRNAs, the qPCR can also detect mutated, yet repaired EBV variants. For all bar diagrams, measurements for (at least) triplicate experiments + STD are presented.

Since EBV can serve as a target for CRISPR/Cas9 genome editing, we reasoned that targeting of viral genetic factors critically involved in EBV genome maintenance could render the virus unstable, causing its loss from latently infected cells. We designed gRNAs targeting the viral EBV nuclear antigen 1 (EBNA1) and several areas within the EBV origin of replication (OriP). The latter included EBNA1 binding sites and the dyad symmetry element, all of which are involved in EBV episome maintenance and replication [37, 38]. As a model system we used the Burkitt’s lymphoma Akata-Bx1 cells that carry a recombinant EBV expressing eGFP under control of the CMV promoter [39]. Hence, eGFP expression serves as a marker for the presence of EBV in these cells. Upon transduction of the Akata-Bx1 cells with gRNAs targeting EBNA1, we observed a loss in eGFP expression in 40–60% of the cells, which did not occur in control cells expressing gRNAs targeting cellular genes (Fig 2A and 2B). Also targeting the EBNA1 binding sites within the EBV OriP and the dyad symmetry element induced a clear loss in eGFP expression from Akata-Bx1 cells (Fig 2A and 2B), suggesting depletion of EBV from these cells. Sequential introduction of combinations of these active gRNAs induced an almost complete loss of eGFP from the majority of cells (Fig 2B, right bar diagrams). We assessed the EBV content in these gRNA-expressing Akata-Bx1 cells by qPCR and indeed detected a strong reduction of the EBV genome content in the cells carrying double gRNAs (Fig 2C). Targeting EBNA1 with two different gRNAs proved most efficient, inducing over 95% loss of EBV genomes. These results indicate that CRISPR/Cas9-mediated targeting of essential regions within the EBV dsDNA effectively reduces the viral genome content in latently infected cells.

### CRISPR/Cas9 targeting of essential HCMV protein-encoding genes impairs virus replication

**Fig 3:**
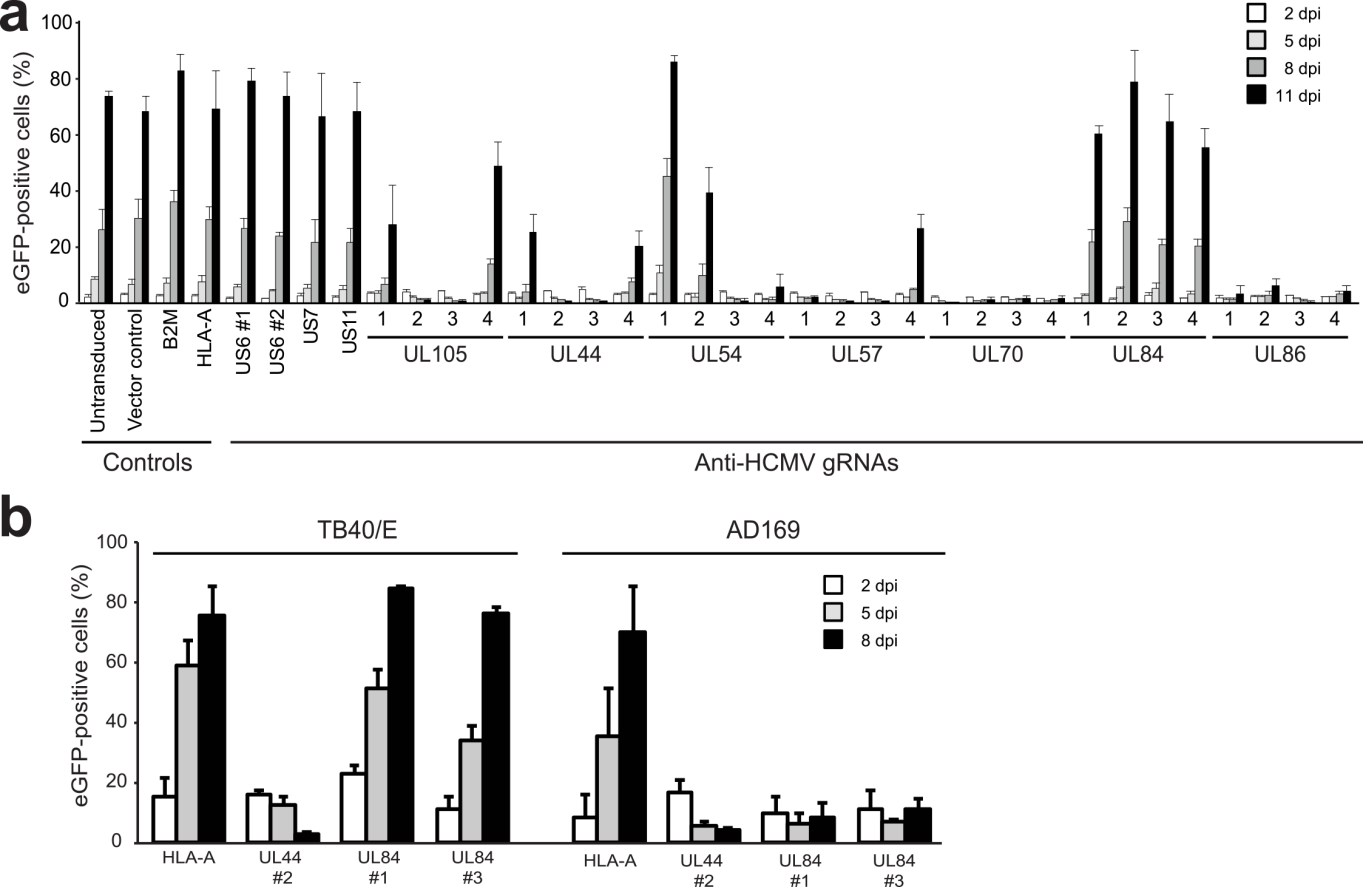
Anti-HCMV gRNAs efficiently abrogate HCMV replication in human cells. **a)** Targeting essential HCMV genes with CRISPR/Cas9 impairs HCMV replication. MRC5 cells transduced with the indicated gRNAs were infected with HCMV-eGFP strain TB40/E at an MOI of 0.05 and subjected to flow cytometry at 2, 5, 8, and 11 dpi to assess the percentage of eGFP-positive infected cells. For each essential gene, four different gRNAs were monitored. Besides targeting human genes as controls, gRNAs targeting nonessential HCMV genes US6, US7, and US11 were included. **b)** Anti-HCMV gRNAs impair replication of both TB40/E and AD169 strains with the exception of anti-UL84 gRNAs. gRNA-expressing MRC5 cells were infected with eGFP-tagged TB40/E or AD169 and the percentage of eGFP-expressing cells was monitored at 2, 5, and 8 dpi. For bar diagrams in a) triplicate measurements + STD are presented. Bar diagrams in b) are from (at least) duplicate measurements.

Since CRISPR/Cas9 proved efficient in editing and clearing latent EBV infections, we next assessed whether we could impact herpesvirus replication in human cells. For this, we turned to a lytic infection model for HCMV, the most-commonly studied member of the *Betaherpesvirinae*. Replication of HCMV is dependent on a large number of viral replication factors. We selected seven of these essential genes and asked whether CRISPR/Cas9 targeting of these genes impact virus infection. We designed four gRNAs per gene for the viral polymerase *UL54*, the polymerase accessory protein *UL44*, the single strand DNA binding protein *UL57*, the primase *UL70*, the DNA helicase *UL105*, the major capsid protein *UL86*, and *UL84*, which is involved in the initiation of viral DNA replication [40, 41]. After lentiviral delivery of the gRNAs to MRC5 cells, the cells were challenged with eGFP-encoding HCMV derived from the TB40/E strain [42]. For each of the essential HCMV genes, one or more gRNAs were capable of almost completely impairing HCMV replication which resulted in survival of the cells and absence of eGFP expression as assessed by flow cytometry (Fig 3A). Unexpectedly, none of the gRNAs targeting UL84 were effective at limiting infection (Fig 3A). In cells transduced with control vector or vectors carrying gRNAs targeting host genes, the percentage of eGFP-positive cells was similar as observed for untransduced cells (Fig 3A). The gRNAs targeting the nonessential HCMV genes *US6*, *US7*, and *US11* also did not interfere with HCMV replication. We assessed the percentage of HCMV sequences that were edited at the US7 and US11 target sites via CRISPR/Cas9 engineering, and observed alteration at these sites in 76 and 95% of cases, respectively. This shows that, although these genes were mutated, this did not interfere with virus replication.

The inhibitory capacity of the anti-HCMV gRNAs was also assessed towards a different strain of HCMV: AD169. Most gRNAs that were effective at limiting TB40/E replication also impaired AD169 replication (Fig 3B). Unlike TB40/E, AD169 was targeted successfully by two independent gRNAs directed against *UL84* (Fig 3B). Hence, replication of AD169 crucially depends on UL84, whereas replication of TB40/E does not. This data agrees with experiments described by Spector and Yetming [43].

In conclusion, effective inhibition of HCMV replication can be achieved using the CRISPR/Cas9 technology; this requires targeting of essential HCMV genes.

### Inefficient targeting of HCMV by single gRNAs selects for viral escape mutants after prolonged replication

**Fig 4:**
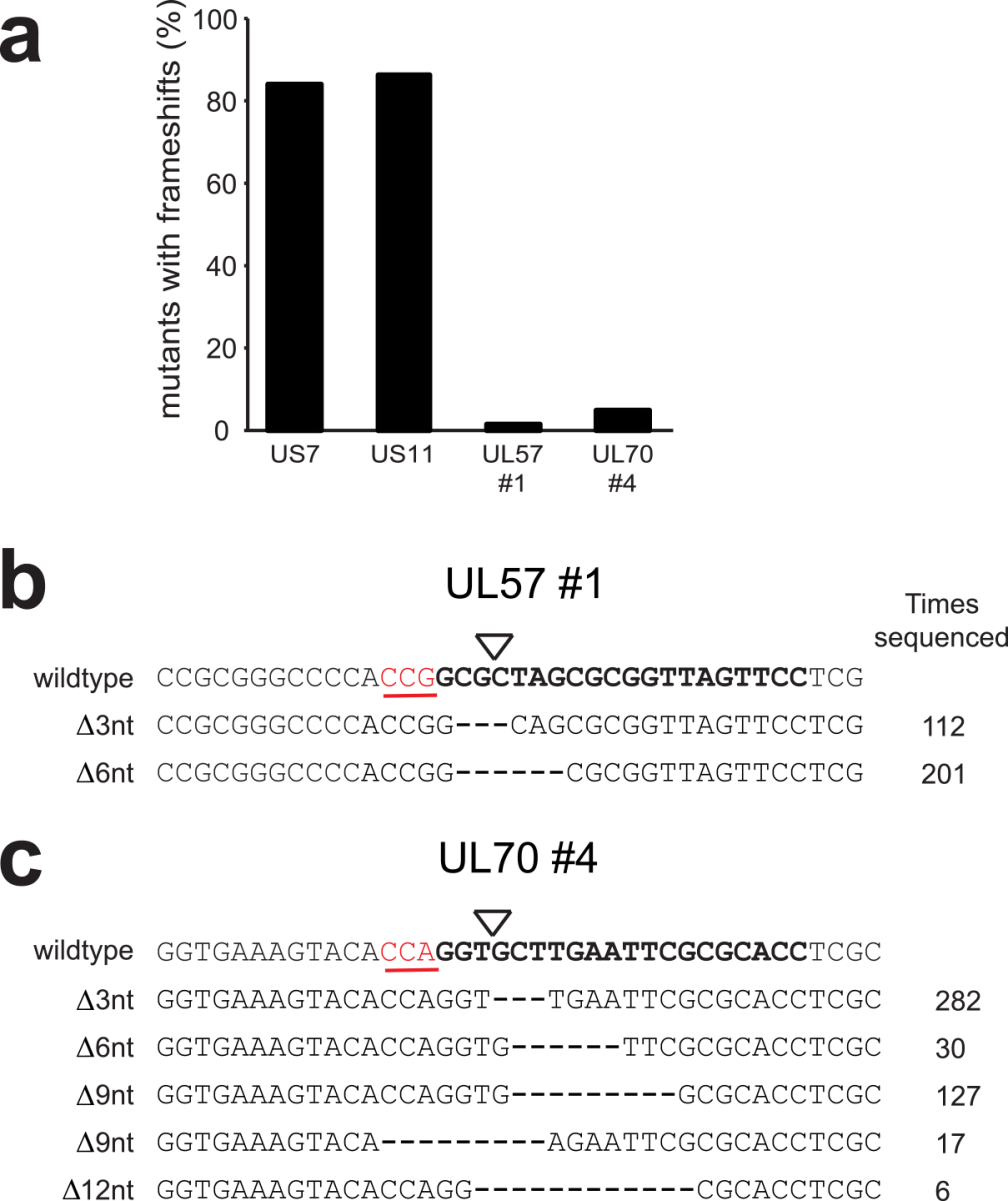
Targeting of HCMV by single gRNAs directed against essential genes selects for viral escape mutants after prolonged culture. **a)** Sequence analysis of HCMV-eGFP variants present after 21 days of culturing in anti-UL57 and UL70 gRNA-expressing cells. The sequences of mutant variants were assessed and the percentage of these that contain in frame-shift mutations at the target site are presented. Genomic DNA was amplified by PCR and subjected to 454 sequencing. Sequences of HCMV variants upon CRISPR/Cas9 targeting of the nonessential HCMV genes US7 and US11 were derived at two days post infection. Number of sequences analyzed: 333 for US7, 331 for US11, 319 for UL57, and 541 for UL7. Bar diagrams are derived from a single 454 sequencing experiment. **b and c)** Commonly sequenced variants are depicted for UL57 #1 and UL70 #4. The gRNA-target site is displayed in bold, the PAM sequence as red underlined text, and the cleavage site as a triangle. The number of reads per variant is indicated.

Many single anti-HCMV gRNAs effectively impaired HCMV infection, preventing virus replication and spread up to 11 days post infection (Fig 3A). However, replication-competent virus emerged after prolonged culture of cells in the majority of gRNA expressing cells. This may be due to the outgrowth of virus variants that harbor CRISPR/Cas9-induced mutations that still allow for expression of functional proteins. To assess this, virus variants were isolated from two cultures of CRISPR-expressing cells that were infected at high MOI (0.5) and displayed outgrowth of virus after 21 days of culture (UL57 gRNA #1 and UL70 gRNA #4). Subsequent 454 sequencing of the targeted genes predominantly detected variants that maintained the correct reading frame of these two essential genes (Fig 4) as mostly deletions of complete codons (i.e. 3-6-9-12 bp, etc) were detected. We quantified the number of sequences with frameshift variants, and observed a clear depletion of these in the anti-HCMV gRNA expressing cells targeting essential genes (1,3% for UL57 #1, and 4,6% for UL70 #4) as compared to gRNAs targeting the nonessential genes US7 and US11 (83,5 and 85,8% respectively) (Fig 4A). Furthermore, the sequence complexity of the mutants selected upon UL57 and UL70 targeting was low (Fig 4B and 4C), suggesting selection of few ‘fit’ variants and subsequent expansion of these infectious mutants over time. To conclude, the CRISPR/Cas9-technology represents a promising strategy to impair HCMV replication; however, its successful application requires effective modification of the viral genome to avoid the emergence and selection of escape variants that bypass CRISPR/Cas9 editing.

### CRISPR/Cas9 targeting of essential HSV-1 protein-encoding genes impairs virus replication

**Fig 5:**
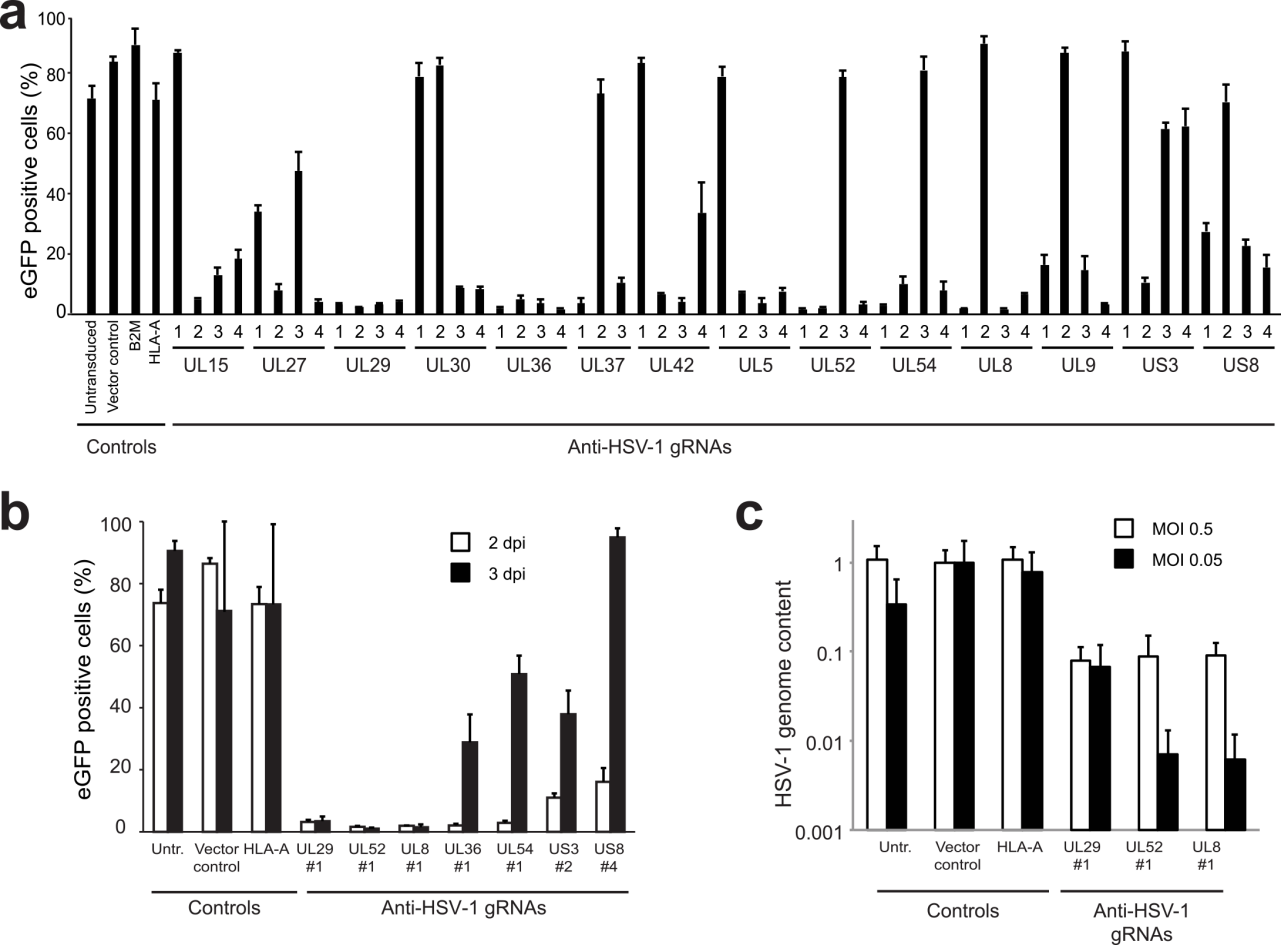
Anti-HSV-1 gRNAs impair HSV-1 replication. **a)** Vero cells were transduced with the indicated gRNAs and subsequently infected with HSV-1-eGFP at an MOI of 0.05. To assess the percentage of virus-infected cells, eGFP-expression was analyzed by flow cytometry at 2 dpi. Four gRNAs/gene targeting twelve essential HSV-1 genes and two non-essential genes (US3 and US8) were assessed. As controls, empty vector-transduced cells and gRNAs targeting the human genes *HLA-A* and *B2M* are presented. **b)** Prolonged inhibition of HSV-1 replication by gRNAs targeting essential genes UL8, UL29, and UL52. Select gRNA-expressing cells from a) were monitored for HSV-1-eGFP presence at 2 and 3 dpi. **c)** Indicated gRNA-expressing cells were infected with HSV-1-eGFP at MOI 0.5 or 0.05 and the amount of HSV-1 viral genomes present in the supernatant was assessed by qPCR at 4 dpi. The relative HSV-1 genome content was normalized to supernatant harvested from cells transduced with vector control. For all bar diagrams, measurements for triplicate experiments are presented + STD.

Since CRISPR/Cas9 proved efficient in limiting productive infection of the slowly replicating HCMV virus, we next assessed whether also the fast replicating alphaherpesvirus HSV-1 can be inhibited using this approach. We designed four gRNAs/gene targeting twelve essential HSV-1 genes [44]: the terminase *UL15*, the glycoprotein B coding *UL27*, the major binding protein *UL29*, the DNA polymerase *UL30*, the tegument protein *UL36*, the capsid assembly protein *UL37*, the DNA polymerase processivity factor *UL42*, the DNA replication protein *UL5*, the DNA helicase/primase complex protein *UL52* and associated protein *UL8*, the transcriptional regulation protein *UL54*, and the replication origin binding protein *UL9*. We included gRNAs targeting the nonessential protein kinase *US3* and the surface membrane protein *US8*. These anti-HSV-1 gRNAs were introduced into Vero cells. Subsequently, the cells were infected with HSV-1-eGFP, and monitored for eGFP expression as a measure for HSV-1 infection after 2 days. High percentages of infected cells were detected in untransduced cells and in cells transduced with empty vector or with vectors carrying gRNAs directed against cellular genes (Fig 5A). Most gRNAs targeting essential HSV-1 genes impaired virus replication effectively. In contrast to our observations for HCMV, targeting of nonessential HSV-1 genes (US3 and US8) also reduced HSV-1 replication, albeit to a lesser extent as compared to targeting essential genes.

Infection of gRNA-expressing Vero cells with HSV-1 resulted in breakthrough of HSV-1 for most gRNAs at 3 dpi (Fig 5B). Interestingly, the gRNAs targeting UL29, and the primase-helicase complex genes UL8 and UL52 [45, 46] maintained their potency at 3 dpi. This effect was apparent at both high (0.5) and low MOI (0.05) and resulted in a concomitant loss of HSV-1 genomes from infected cells (Fig 5C). These results indicate that infection with the fast replicating HSV-1 can be limited considerably; this, however, requires the use of effective gRNAs.

### Simultaneous targeting of HSV-1 with two gRNAs completely impairs replication

**Fig 6:**
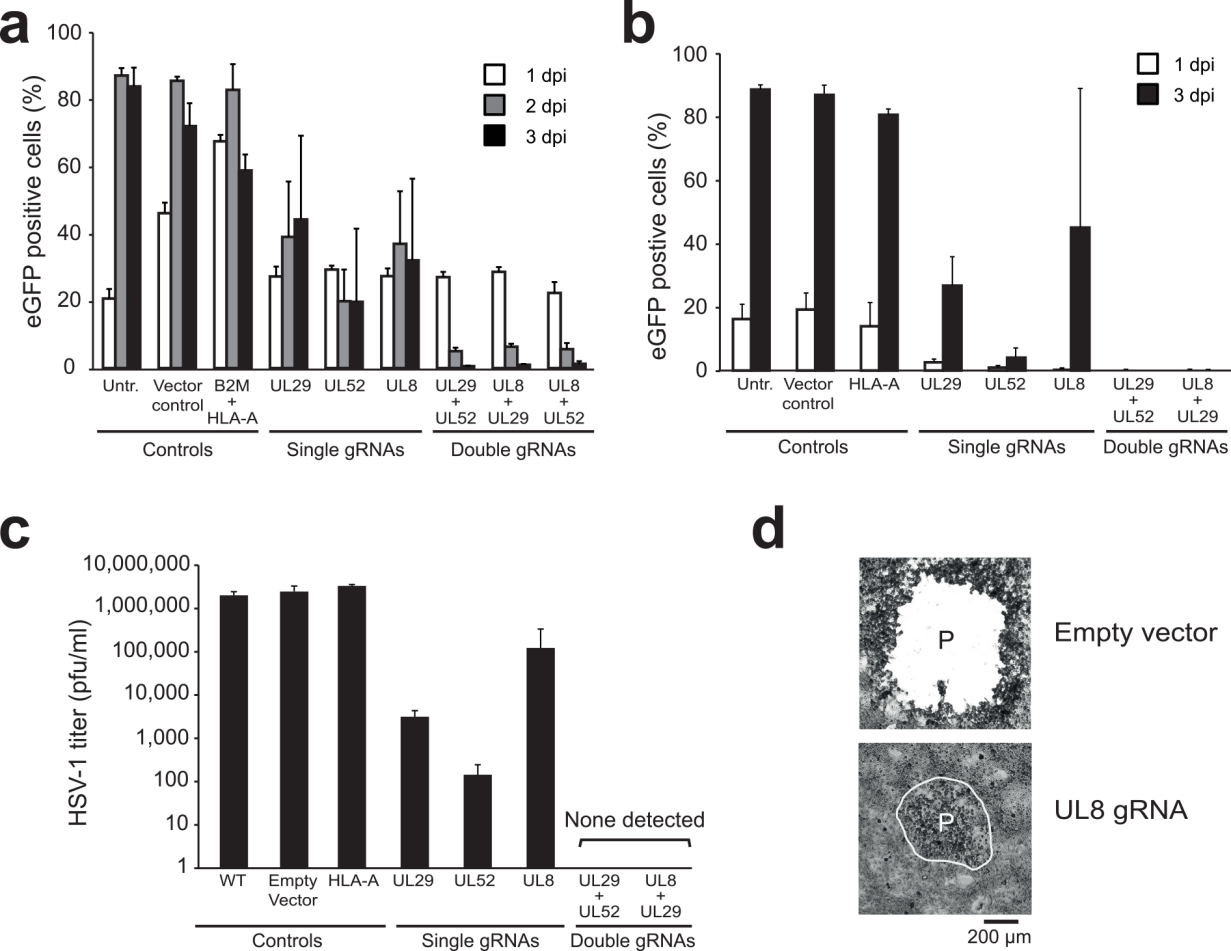
Simultaneous targeting of HSV-1 with two gRNAs completely impairs virus replication. **a)** Vero cells were transduced with the indicated single or double gRNAs and subsequently infected with HSV-1-eGFP at an MOI of 0.5. Cells were analyzed for eGFP-expression by flow cytometry at 1, 2, and 3 dpi to assess the percentage of virus-infected cells. **b)** Similar experiment as in a) but now performed in human MRC5 cells and at an MOI of 0.005, as MRC5 cells are more susceptible to HSV-1 infection. Cells were analyzed for eGFP-expression by flow cytometry at 1 dpi and 3 dpi to assess the percentage of successfully infected cells. **c)** Supernatants from b) were subjected to plaque assays to quantify the infectious HSV-1 titer produced by gRNA-expressing MRC5 cells that had been infected with HSV-1-eGFP three days earlier. Plaques were scored if visible by eye. **d)** Plaques from c) obtained after infection of cells with HSV-1 harvested from control or anti-*UL8* gRNA-expressing MRC5-cells were analyzed by light-microscopy. Whereas large plaques (‘P’) are observed in infected cells not carrying any gRNAs, virus harvested from UL8 gRNA- expressing cells induced microplaques. In double gRNA-treated cells, no signs of infection were observed. For all bar diagrams, measurements of triplicate experiments are presented + STD.

When using single gRNAs, prolonged propagation of cells infected with herpesviruses at high MOIs may result in the selection of escape mutants that bypass CRISPR/Cas9 editing. Simultaneous targeting of multiple essential viral genes may impair viral replication more effectively and prevent the generation of escape mutants. To investigate this possibility, Vero cells expressing single gRNAs targeting the essential HSV-1 genes UL8, UL29, or UL52 were compared to cells carrying double gRNAs targeting combinations of these genes. Cells expressing single gRNAs were partially protected from challenge with HSV-1 at high MOI, but the cells were not able to clear HSV-1 at 3 dpi (Fig 6A). Expressing double anti-HSV-1 gRNAs, however, induced a gradual loss of HSV-1 infected cells and resulted in a cell population that was HSV-1 negative at 3 dpi (Fig 6A). Similar results were obtained when double anti-HSV-1 gRNAs were introduced in human MRC5 cells that were challenged with HSV-1 (Fig 6B). To monitor the impact of the gRNAs on the generation of HSV-1 progeny, we assessed virus titers in the supernatants of these single and double gRNA-expressing cells via plaque assay (Fig 6C and 6D). As expected, we observed a clear drop in the amount of infectious viral particles present in the supernatants of these gRNA-expressing cells. A single gRNA targeting UL52 resulted in a four-log reduction in virus titer (Fig 6C). Combining two gRNAs targeting UL29 and UL8 or UL29 and UL52, however, caused a complete loss of infectious viral particles, with more than six log reduction in progeny virus (Fig 6C). In the plaque assay performed with supernatants harvested from single gRNA-expressing cells, smaller ‘plaques’ appeared that were not visible by eye. This was especially apparent for viruses harvested from the anti-UL8 gRNA-expressing cells (Fig 6D, lower panel). We speculate that the reduced plaque size was caused by mutant, attenuated virus. Importantly, we did not observe any signs of infection in the plaque assay performed with supernatants harvested from double gRNA-expressing cells. Therefore, our results show that simultaneous targeting of multiple essential viral genes using CRISPR/Cas9 greatly improves the efficiency at which herpesviruses are cleared from infected cells as compared to single gene targeting.

### No detectable off-target editing at predicted off-target sites within the human genome

Since prolonged expression of Cas9 and gRNAs in human cells could result in editing at off-target sites, we analyzed the activity of nine gRNAs to the top three predicted off-target sites within the human genome. We PCR-amplified these 27 loci from gRNA-expressing and control cells and subjected these to 454 sequencing (S3 Table) or conventional Sanger sequencing (S2 Fig). Importantly, no signs of CRISPR/Cas9-induced editing at these loci were detected, suggesting that overt editing at undesired sites did not occur.

### Anti-HSV-1 CRISPR gRNAs abrogate replication of HSV-1 reactivated from quiescence

**Fig 7:**
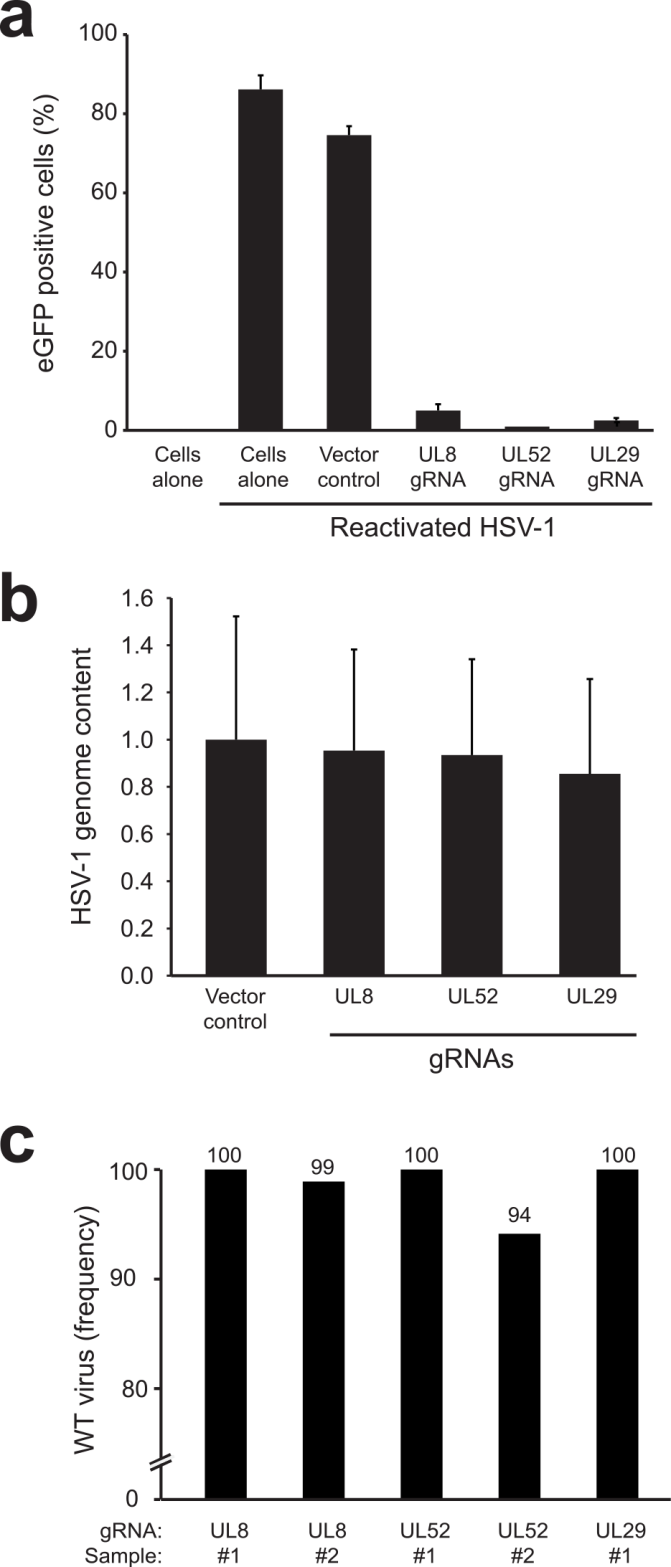
Anti-HSV-1 CRISPR gRNAs are ineffective in targeting quiescent HSV-1 but do abrogate replication of reactivated HSV-1. **a)** MRC5 cells exogenously expressing Cas9 were infected with quiescent HSV-1-eGFP. Anti-HSV-1 gRNAs were introduced into the MRC5-Cas9 cells via lentiviral transduction and cells were subsequently superinfected with HCMV to reactivate latent HSV-1. The percentage of cells with replicating HSV-1 were assessed 3 days post HCMV superinfection by flow cytometry. Two quiescent control (‘cells alone’) samples are presented: one not superinfected with HCMV to assess spontaneous HSV-1 reactivation levels, and one superinfected with HCMV to assess the reactivation potential and subsequent HSV-1 replication in these cells. Control vector corresponds to empty gRNA-vector. **b)** Relative amount of HSV-1 genomes in quiescent HSV-1-eGFP cells transduced with the indicated gRNAs as assessed by TaqMan qPCR. DNA input was normalized to RNAseP levels. The relative HSV-1 content was compared to quiescent MRC5 cells transduced with empty vector control lentivirus. **c)** Analysis of anti-HSV-1 genome editing by next generation sequencing. The percentage of WT sequences at the indicated target sites is presented upon introduction of the corresponding gRNA. For UL8 and UL52, two samples were analyzed. In control vector treated quiescent HSV-1 cells, no CRISPR/Cas9 editing was observed at these target sites (gRNA specific indels <0.1%). CRISPR/Cas9 genome editing was only observed for UL52 #2 and UL8 #2 where CRISPR/Cas9 editing was apparent in ±6 and ±1% of sequences. The nature of these mutations is presented in S4 Fig.

Next, we assessed whether the CRISPR/Cas9 system can target the latent state of HSV-1 in infected cells. As a model, we adapted the *in vitro* HSV-2 quiescence model previously established by Russell and Preston [47, 48]. In short, MRC5 human lung fibroblast cells were infected with HSV-1-eGFP and immediately incubated at 42°C for 4 days. At this temperature, HSV-1 replication is non-permissive and the virus establishes a quiescent state resembling latency. Upon subsequent incubation of the culture at 37°C, HSV-1 remains in a quiescent stage for a prolonged period of time (S3A Fig). Upon subsequent superinfection with AD169 HCMV, HSV-1-eGFP reactivates from quiescence resulting in virus spread to neighboring cells which can be monitored by assessing CPE by microscopy or eGFP expression by flow cytometry (S3A and S3B Fig). Although the MRC5 cells contain quiescent HSV-1, the cells themselves are not quiescent and maintain the ability to divide. As there is a gradual loss of quiescent HSV-1 prior to reactivation caused by cell division, the timeframe to study the effect of anti-HSV-1 gRNAs is limited. Therefore, to increase lentiviral titers carrying anti-HSV-1 gRNAs, we expressed Cas9 from a separate lentiviral vector and generated an MRC5 line stably expressing the endonuclease (MRC5-Cas9 cells). Upon establishment of a quiescent HSV-1 infection in these cells, anti-HSV-1 gRNAs were introduced and selected to purity. We next assessed the effect of these gRNAs on HSV-1 reactivation by superinfection with HCMV. Untreated MRC5 cells containing quiescent HSV-1 showed no signs of HSV-1-eGFP replication (Fig 7A, cells alone), whereas superinfection of these cells with HCMV resulted in reactivation of HSV-1-eGFP and subsequent virus replication and spread (Fig 7A, cells alone, reactivated HSV-1). Quiescent HSV-1 was also efficiently reactivated in control cells transduced with empty gRNA vector, resulting in rapid spread of HSV-1-eGFP, (Fig 7A, vector control, reactivated HSV-1). However, cells expressing anti-HSV-1 gRNAs targeting UL8, UL52, or UL29 displayed abrogated HSV-1-eGFP replication.

The observed block in HSV-1-eGFP replication upon reactivation may be caused by direct targeting of the quiescent HSV-1 genome by the CRISPR/Cas9 system. The latter could result in destabilization of the quiescent genome or mutagenesis of essential genes. To assess the mechanism of the observed interference with HSV-1 replication, we isolated genomic DNA from gRNA-expressing quiescent MRC5-Cas9 cells prior reactivation and quantified their HSV-1 genome content by qPCR. No evident loss of HSV-1-eGFP genomes was observed in anti-HSV-1-gRNA expressing cells as compared to control cells (Fig 7B). We next assessed whether CRISPR/Cas9-induced editing of the quiescent HSV-1 genomes occurred at the gRNA target sites. For this, we PCR-amplified and deep-sequenced the gRNA target sites from quiescent cells in the presence or absence of anti-HSV-1 gRNAs (Fig 7C). No indels were present in any of the 5 lines receiving empty vector gRNA controls. Minor editing frequencies were detected in 2 out of 5 gRNA-expressing samples where 6 and 1% of sequences contained indels in a UL52 and UL8 gRNA-expressing line respectively (Fig 7C and S4 Fig). Intriguingly, most of the mutant UL52 reads were from a single 21 in-frame deletion that could result in functional UL52 protein, suggesting that it has been actively selected for. As no selection occurs during quiescence due to absence of virus replication, we speculate that the low percentage of CRISPR/Cas9 indel formation was not caused by targeting of the quiescent genome, but rather by editing of newly produced viral genomes in sporadic spontaneously reactivated cells. Indeed, the potent effect of gRNAs on reactivated HSV-1-eGFP (Fig 7A) cannot be explained by the ineffective indel formation measured in Fig 7C and points towards editing of newly produced viral genomes.

To conclude, our data suggest that anti-HSV-1 gRNAs can actively restrict replicating virus, but, in the model used, are inefficient at targeting the HSV-1 genome during quiescence when directing gRNAs to the genomic regions of UL8, UL29, and UL52.

## Discussion

Herpesviruses such as HSV-1, HSV-2, HCMV and EBV are a frequent cause of disease and treating these herpesvirus infections remains a major challenge. Current therapies are mainly geared towards limiting productive infections by targeting virus replication via antiviral drugs such as the guanosine analogues acyclovir and ganciclovir. Although these compounds effectively inhibit the viral DNA polymerase during replication, they have limited impact on the latent stage of herpesvirus infections that relies on host polymerases for viral genome maintenance in dividing cells [7]. Strategies to impact the latent stage of infection often depend on reactivation of a latent infection by using chemotherapeutic agents and subsequent targeting of these cells by e.g. ganciclovir [49]. Such strategies are not always effective and may result in loss of the target cell. Although this approach may be beneficial to clear EBV-driven cancer cells, other instances ask for sparing of the infected cell, for example in HSV-1, HSV-2 and VZV-infected neurons. Hence, there is a need for a direct and potent strategy to limit herpesvirus infections by specifically targeting the viral genome or its maintenance. Preferably, such approaches should impact both lytic and latent herpesvirus infections. Indeed, recently, the HSV-1 genome has been targeted by using meganucleases [50, 51]. Here, we performed proof-of-concept studies to show that the CRISPR/Cas9 genome engineering system can be efficiently used to limit or eradicate three of the most prevalent herpesviruses from human cells: HSV-1, HCMV and EBV.

EBV relies on EBNA1 for maintenance of the viral episome. EBNA1 is a well-studied protein that is consistently expressed in EBV-infected cells, including all EBV-associated malignancies [38, 52–54]. Indeed, selective EBNA1 inhibition via siRNAs [55], small molecule inhibitors [56, 57], or dominant-negative forms of the protein [58] impairs EBV latency and results in a loss of EBV episomes from infected cells. In the present study, we show that the CRISPR/Cas9 system can indeed target the EBNA1 protein-coding sequence or regulatory EBNA1 binding sites on the EBV genome, as a means to inhibit EBV episome maintenance. We show that the CRISPR/Cas9 system can target and edit EBV genes in naturally EBV-transformed cells of lymphoid and epithelial origin. Taken into account that the gastric carcinoma cell line SNU719 carries ±800 EBV genomes/cell [59], the efficiency of editing was high. Indeed, targeting of the essential EBNA1 gene using a single gRNAs in Akata-Bx1 cells resulted in 50–60% loss of latent EBV from cells. However, a proportion of the cells still contained EBV, which could be either wt unedited EBV, or mutant EBV that has received CRISP/Cas9-mediated mutations at the target region while retaining EBNA1 activity (e.g. by in an in-frame deletion). Subsequent introduction of a second gRNA targeting a different region on the EBNA1 gene, however, further enhanced the removal to up to 95% of EBV genomes. We hypothesize that simultaneous administration of multiple anti-EBV gRNAs into latently infected cells will further enhance the destabilization of EBV episomes and may facilitate complete eradication of the virus. This clearance could result in the loss of EBV-driven tumorigenic, cell cycle-promoting functions, anti-apoptotic features, and anti-inflammatory functions of EBV-encoded gene products and as such may serve as a new therapeutic strategy to combat EBV-associated malignancies. Indeed, an independent study showed that transient transfection of multiple anti-EBV gRNAs cleared EBV from a small subset of latently infected Raji B-cells, thereby inducing a proliferation arrest in these cells [29]. Our approach relies on stable expression of anti-EBV gRNAs via lentiviral vectors and induces an almost complete loss of EBV episomes, suggesting that a further optimization of this approach may allow for the complete removal of EBV from human cells.

CRISPR/Cas9 targeting of essential HCMV and HSV-1 genes efficiently abrogated virus replication in human cells. The mechanism of protection by CRISPR gRNAs is likely threefold. Introducing a dsDNA break in the viral genome may impair packaging of intact viral genomes, limiting the production of viral particles. Second, a dsDNA cut within an essential gene may impair the production of the targeted protein, and hence impact the biology of virus replication. Third, upon repair of the CRISPR/Cas9-induced dsDNA break by the hosts’ non-homologous end joining machinery, the target site is frequently mutated, resulting in the formation of virus mutants with (often) impaired protein function. In the latter case, the generation of new viral particles may be hampered directly, or mutated DNA may be properly packaged into new viral particles, but replication is subsequently halted in newly infected cells. In all three cases, the virus replication will be impaired. Indeed, for HSV-1 we observe a block in virus replication when we target either essential or nonessential genes. This indicates that a dsDNA cut within the HSV-1 genome alone is sufficient to impact virus replication, likely because less correctly assembled viral particles are generated, thereby blocking subsequent infection of naïve cells. By targeting essential HSV-1 genes, we observe an increased impairment of HSV-1 replication. Here, the effect is likely caused by a combination of genome destabilization and impaired expression of the essential genes. The latter effect is further enhanced by simultaneous targeting of two essential genes using two gRNAs. Here, the viral genome may be fragmented, which adds to the potency observed for single anti-HSV-1 gRNAs. Indeed, when targeting two sites in a given linear piece of DNA, large parts can be excised out of human [17, 60], dsDNA viral [23, 24, 29, 30, 61], and integrated HIV genomes [32–36]. Intriguingly, we did not observe reduced virus replication when targeting the nonessential HCMV genes US7, US10, or US11, whereas targeting essential genes did. This result was unexpected, and may be caused by a difference in replication kinetics between HCMV and HSV-1. Since HCMV is a slowly replicating virus, the DNA repair machinery may have sufficient time for complete repair of the cleaved viral genomes to occur. Since *in vitro* replication of the mutant HCMV is not affected by mutations in nonessential genes, the virus may replicate as efficiently as the wild type virus. However, for fast-replicating viruses such as HSV-1, the replication speed could outpace the time needed for complete dsDNA break repair. Indeed, nonhomologous end-joining (NHEJ) kinetic studies [62] showed that ±75% of dsDNA breaks in MRC5 cells are repaired within a two-hour timeframe, whereas complete repair requires approximately 24 hours to occur. Since HSV-1 has a replication cycle of less than 18 hours [63], it is possible that slow NHEJ kinetics impacts HSV-1 more profoundly than HCMV that has a replication cycle of ±72 hours [63].

Although the majority of gRNAs targeting essential genes of HSV-1 and HCMV were effective in abrogating virus replication, some showed limited to no activity (Figs 3A and 5A). Indeed, CRISPR/Cas9 activity can vary greatly between different gRNA sequences targeting the same gene [64]. The potency of gRNAs is partially dependent on sequence features of the expressed gRNA as well as the composition of the PAM sequence and downstream nucleotides in the target allele [64]. Additionally, local chromatin structure at the genomic target site impacts the ability of Cas9 to bind to DNA [65, 66], and thereby affect CRISPR/Cas9 activity. Since HSV-1 is a fast replicating virus, low activity anti-HSV-1 gRNA are likely ineffective in abrogating virus replication and spread as unaltered HSV-1 virus replication will quickly outcompete mutant viruses.

Using single gRNAs targeting genes essential for HCMV or HSV-1, we were able to severely impair replication of these viruses in human cells. Using a double gRNA–expression approach, the generation of new infectious HSV-1 was completely blocked, which caused a >10^6^ drop in virus titers. We did observe the emergence and selection of escape mutants upon single gRNA treatments targeting essential genes for both HCMV and HSV-1. The HCMV mutants were derived from CRISPR/Cas9-induced genome editing events, as all selected variants displayed editing at the gRNA target cleavage site. There was a strong selection for variants that retained the reading frame of the targeted genes, as >95% of identified variants contained deletions of complete codons (changes of 3-6-9-12 bp etc) at the target site, whereas for gRNAs targeting the nonessential US7 and US11 genes such edits occurred at much lower frequencies (±15%). The ‘in frame’ variants likely retained (partial) functionality of the targeted proteins and were hence selected for during the infection cycle. Indeed, in the plaque assay performed with supernatants harvested from anti-UL8 gRNA-expressing cells, we noted the presence of small plaques indicative of presence of infectious, yet attenuated HSV-1 variants. Importantly, we did not observe any outgrowth of escape mutants when we targeted two essential HSV-1 genes. Apparently, the impact of HSV-1 genome fragmentation together with the loss of essential viral gene expression is too stringent to allow for the formation of infectious virus variants that bypass CRISPR/Cas9 targeting. Further increasing the number of anti-viral gRNAs expressed within a single cell may enhance this protective effect.

We observed broadly similar potencies of anti-HCMV gRNAs towards two commonly used HCMV strains (AD169 and TB40/E). However, CRISPR/Cas9 targeting of the HCMV UL84 gene protected human cells from infection by the AD169 strain, but not by the TB40/E strain. Previously, it has been shown that replication of HCMV strain TB40 is indeed UL84-independent [43]. If the CRISPR/Cas9 system is to be applied to combat human herpesviruses in a clinical setting, it would be important to target viral genes that are essential for all clinical virus isolates. Also, since the CRISPR/Cas9 system acts on dsDNA, directing gRNAs to well-conserved regions in these genes is essential to target the majority of viral strains. Nevertheless, CRISPR/Cas9 target cleavage does allow few mismatches between the target DNA and the gRNA [17], allowing also less conserved regions to be targeted. In our studies, we targeted gRNAs to the N-terminal coding regions of essential viral genes. In this approach, indel mutations causing frame-shifts will completely impair protein function. An alternative strategy to further enhance the potency of anti-viral CRISPRs is to target gRNAs to DNA coding for key amino acid residues within such essential proteins. This way, any substitution or indels at these sites could render the targeted protein non-functional and prevent the emergence of escape mutants. Unlike fast-evolving RNA viruses such as HIV and Influenza virus [67], dsDNA herpesviruses evolve at a slow rate [68]. Therefore, the emergence of escape mutants will be mainly governed by the stochastic process of CRISPR/Cas9-induced error-prone NHEJ, and not by mutagenesis events caused by the replication machinery. Indeed, the likelihood of generating in-frame indels at a target site is relatively high (±15% for HCMV US7 and US11 genes) resulting in a significant chance for selecting mutant virus that retain fitness. Thus, instead of using single gRNAs to target herpesviruses, simultaneous administering of multiple CRISPR gRNAs will further reduce the likelihood of generating escape mutants as the viral genome will not only be mutagenized at multiple essential sites, but will also become fragmented

The potential of the CRISPR/Cas9 system to induce off-target editing of the human genome has been a matter of concern [15, 69]. Even though our CRISPR-expressing cells continuously expressed the CRISPR/Cas9 system for several weeks, we did not identify any obvious off-target activity at the top three predicted off-target sites for the nine most potent gRNAs. However, it remains possible that these gRNAs do induce editing at other genomic sites. Clearly, if the CRISPR/Cas9 system would be applied to combat herpesviruses in a clinical setting, the potential off-target activity should be reduced to an absolute minimum. Much progress has been made to limit CRISPR/Cas9 off-target activity. For example, Ran *et al*. describe the use of paired gRNAs in combination with a Cas9 mutant that can only induce nicking in the dsDNA. NHEJ will only occur when two successful gRNA-directed cuts occur in close proximity [70]. Another study describes the use of tru-gRNAs, where shortened gRNAs retain their potent on-target activity, but show much reduced off-target editing [71]. A combination of these two approaches may further reduce the (already limited) off-target activity of the CRISPR/Cas9 system. Another strategy is to engineer Cas9 derivatives by mutagenesis that have reduced off-targeting potential, yet retain their on-targeting activity [72–74]. Alternatively, as many Cas9 endonuclease variants exist in nature, it is likely that new variants will be identified that hold potent on-target, yet low off-target activity. A combination of these approaches will further reduce the (already limited) off-target activity of the CRISPR/Cas9 system.

Our data show that the CRISPR/Cas9 system can efficiently target latent EBV, but not quiescent HSV-1 in the model used. Although we did identify minor CRISPR/Cas9-mediated editing of quiescent HSV-1 in MRC5 cells in 2 out of 5 sequencing samples, it remains unknown whether this activity was directed towards the quiescent genome or towards new progeny virus that derived from an early spontaneous reactivation event. The MRC5 quiescence model can display spontaneous reactivation of HSV-1, which results in rapid virus replication and spread. Although we did not detect any signs of spontaneous reactivation in the experiments as presented in Fig 7C, it is conceivable that an early reactivation event occurred, which was subsequently targeted by the expressed gRNA and identified in the deep-sequencing studies. The minor HSV-1 editing that is observed cannot explain the efficient abrogation observed in HSV-1 replication upon HCMV-induced HSV-1 reactivation (Fig 7A). Hence, anti-HSV-1 gRNAs are efficient in targeting actively replicating virus, whereas quiescent HSV-1 genomes are inefficient substrates for the gRNAs used in the model we studied. The mechanisms underlying latency differ for various herpesviruses. This may result in differences in sensitivity to CRISPR/Cas9-mediated editing of latent genomes. EBV resides as an episome in the nucleus of actively dividing cells, where EBNA1 mediates replication of the viral genome via the host replication machinery without the formation of progeny virus [7]. HSV-1 latency on the other hand, occurs in non-dividing cells in sensory neurons where no genome replication occurs [75]. During latency, the HSV-1 genome is heavily methylated and viral gene expression is restricted [75]. It is suggestive that the CRISPR/Cas9 system is inefficient in accessing the tightly repressed state of the HSV-1 quiescent genome, whereas the open structure of the EBV genome during replication is accessible. Indeed, studies using models in human fibroblasts show that the quiescent HSV-1 genome is present in a tightly repressed state which is not responsive to most reactivation stimuli that normally de-repress latent genomes in neuronal cells [76, 77]. Hence, the low activity of CRISPR/Cas9 towards quiescent HSV-1 as observed in our model may not recapitulate the effect of anti-HSV-1 gRNAs *in vivo*. Additional *in vitro* and *in vivo* model systems are needed to assess whether CRISPR/Cas9 can target HSV-1 latency.

Human herpesviruses cause a wide range of diseases. The CRISPR/Cas9 approach may provide an attractive new strategy to combat such complications by directly impairing replication and removing these viral invaders from infected cells. The potential applications are abundant; one could direct anti-EBV gRNAs to remove EBV from EBV-driven tumors as an anti-tumor treatment. Anti-EBV and HCMV gRNAs may be used to deplete virus from tissues prior to transplantation, preventing donor-derived infections in immunocompromised recipients. Upon optimization, anti-HSV-1 gRNAs may be used to target latent HSV-1 in trigeminal ganglia, thereby curing herpes simplex keratitis, a common cause of eye infection leading to corneal blindness. VZV-associated zoster and the related post-herpetic neuralgia, a frequent and serious complication, may be treated in a similar fashion. In all these cases, the development of potent CRISPR/Cas9 delivery systems is of key importance. We have performed our studies using lentiviral delivery vectors; such vectors have been used as delivery vehicle in 114 clinical trials (http://www.abedia.com/wiley/vectors.php) and have proven efficient at correcting genetic defects in humans [78]. Other viral delivery systems may rely on non-integrating adenoviruses or recombinant adenovirus-associated virus (rAAV) as vectors. These non-integrating viruses have been found to effectively deliver a wide-range of genetic factors into human cells and the first rAAV vector has recently been approved for the treatment of lipoprotein lipase (LPL) deficiency in Europe [79, 80]. Especially the neurotropic nature of rAAV vectors [81] make these viruses attractive vehicles to introduce anti-HSV-1, HSV-2 and VZV gRNAs into infected neuronal cells. Furthermore, alternative delivery methods, such as the direct delivery of proteins to cells [82, 83], may remove the risk of insertional mutagenesis.

In conclusion, we have shown that CRISPR/Cas9 technology can effectively clear herpesvirus infections from human cells *in vitro*. We observed highly efficient and specific clearance of EBV from latently infected tumor cells and impairment of HSV-1 and HCMV replication in human cells. By combining two gRNAs targeting two essential HSV-1 genes, we completely inhibited the generation of new infectious virus during a lytic HSV-1 infection *in vitro*. Although CRISPR/Cas9 was inefficient at directing genome engineering of quiescent HSV-1 in our *in vitro* model, virus replication upon reactivation of quiescent HSV-1 was efficiently abrogated using anti-HSV-1 gRNAs. These new insights may allow the design of effective therapeutic strategies to target human herpesviruses during both latent and productive infections.

## Methods

### Cell lines and viruses

MRC5 human lung fibroblast cells, 293T human embryonic kidney cells, and Vero African Green monkey kidney epithelial cells, were obtained from ATCC (American Type Culture Collection). SNU719 cells were kindly provided by Prof. Jaap Middeldorp and were originally obtained from the Korean Cell Line Bank. MRC5 and Vero cells were grown in DMEM (Lonza AG, Switzerland) supplemented with glutamine, penicillin/streptomycin and 10% FCS. 293T and SNU719 cells were grown in RPMI 1640 medium (Lonza AG, Switzerland) supplemented with glutamine, penicillin/streptomycin and 10% FCS.

The EBV-positive Akata-Bx1 cells were kindly provided by Prof. Lindsey Hutt-Fletcher (Louisiana State University Health Sciences Center, Shreveport, USA). Akata-Bx1 cells carry a latent recombinant EBV in which the nonessential thymidine kinase gene has been replaced with a neomycin resistance gene under control of the thymidine kinase promoter and a modified eGFP gene under control of the CMV promoter [39].

The AD169-eGFP recombinant virus was kindly provided by Prof. Robert Kalejta (University of Wisconsin, MA, USA). AD169-eGFP (referred to as AD169 in the text) carries a simian virus 40 early promoter-driven eGFP gene in place of the viral US4-US6 region [84]. The UL32-eGFP-TB40/E strain was kindly provided by Prof. Christian Sinzger, Universitätsklinikum Ulm, Germany). UL32-eGFP-TB40/E (referred to as TB40/E in the text) is a recombinant HCMV expressing eGFP fused to the C terminus of the capsid-associated tegument protein pUL32 (pp150) [42]. HCMV viruses were propagated in MRC5 cells using standard culturing and harvest techniques. HCMV titers were determined in MRC5 cells using the eGFP marker as readout.

The HSV-1-eGFP strain constructed by Prof. Peter O’ Hare (Imperial College, London, UK) was kindly provided by Dr. Georges Verjans (Erasmus Medical Center, Rotterdam, The Netherlands). HSV-1-eGFP is a derivative of HSV-1 strain 17 expressing a VP16-eGFP fusion protein [85]. HSV-1-eGFP virus was propagated in Vero cells using standard culturing and harvest techniques and viral titers were determined in Vero cells using the eGFP marker as readout.

HCMV strain AD169 was obtained from ATCC (VR-538). AD169 virus was used to induce reactivation of latent HSV-1 by superinfection of latent MRC5 cells. AD169 virus was propagated in MRC5 cells using standard culturing and harvest techniques.

### Lentiviral CRISPR/Cas9 vector and other DNA vectors

We constructed a selectable lentiviral CRISPR/Cas vector by altering the lentiviral pSicoR vector [86] (Addgene plasmid 11579, Tyler Jacks Lab, MIT) to express a human codon-optimized nuclear-localized codon-optimized *S*. *pyogenes* Cas9 gene (taken from Addgene plasmid 42229 [17], Zhang lab) that was N-terminally fused to PuroR via a T2A ribosome-skipping sequence [87]. This cassette was expressed from the human EF1A promoter. Additionally, we replaced the mouse U6 promoter with a human U6 promoter which drives expression of a guideRNA (gRNA) consisting of a 18-20bp target-specific CRISPR RNA (crRNA) fused to the *trans*-activating crRNA (tracrRNA) [88] and a terminator sequence. We generated a second variant of this vector, replacing the PuroR gene for a BlastR gene. These vectors are called pSicoR-CRISPR-PuroR and pSicoR-CRISPR-BlastR respectively. The pSicoR-CRISPR-PuroR vector was previously successfully used in Van de Weijer *et al* [89].

crRNA target sequences were designed manually or using the crispr.mit.edu website (Zhang lab, MIT) by entry of the first 250 nucleotides downstream of the start codon for the selected genes. CRISPR gRNA sequences were selected by highest score for specificity and the least off-targets within the human genome, as provided by the online CRISPR design tool. For each virus gene target, multiple independent CRISPR gRNAs were selected and cloned into the pSicoR-CRISPR-PuroR vector and verified by sequencing. gRNA target sequences used in this study are presented as supporting information (S1 Table). For the HSV-1 latency studies, gRNAs were introduced in a variant of the pSicoR-CRISPR-PuroR vector in which the Cas9 gene was removed. In these studies, Cas9 is expressed from the pSicoR-CRISPR-ZeoR vector. This vector is identical to pSicoR-CRISPR-PuroR vector, although PuroR was replaced by ZeoR and the U6-gRNA cassette was removed.

Lentiviral EBV miRNA reporters were cloned in the pSicoR backbone (Addgene plasmid 11579, Tyler Jacks Lab, MIT). For this, the CMV promoter was replaced with a human EF1A promoter that now drives expression of a PuroR-T2A-mCherry cassette allowing for selection and tracking of lentivirally transduced cells. Downstream of the PuroR-T2A-mCherry cassette we cloned single perfect miRNA target sites for the following EBV-encoded miRNAs: BART5-5p (5’-CGATGGGCAGCTATATTCACCTTG-3’), BART6-3p (5’-TCTAAGGCTAGTCCGATCCCCG-3’), and BART16-5p (5’-AGAGCACACACCCACTCTATCTAA-3’). All vectors were sequence verified.

### Generation of CRISPR/Cas9 expressing cell lines

For lentiviral transductions, virus was produced in 24-well plates using standard lentiviral production protocols and third-generation packaging vectors. Due to the large size of the lentiviral pSicoR-CRISPR-PuroR and pSicoR-CRISPR-BlastR vectors, titers were often low. When necessary, lentiviruses were concentrated using Lenti-X (Clontech) ±10 fold prior use. Target cells were transduced with lentiviral supernatants in the presence of polybrene (4μg/ml) via spin infection at 1,000g for 90 minutes at 33 °C. Three days post infection, successfully transduced cells were selected via puromycin (2μg/ml) or blasticidin (10μg/ml) treatment. For Vero cells, we used 5μg/ml puromycin or 20μg/ml blasticidin respectively. Double gRNA expressing cell lines were generated by initial transduction using the appropriate pSicoR-CRISPR-PuroR vector, followed by complete puromycin selection and subsequent transduction with the appropriate pSicoR-CRISPR-BlastR vector and blasticidin selection.

### Primary infections of CRISPR-expressing cells with HCMV-eGFP or HSV-1-eGFP

±35,000 CRISPR expressing cells were seeded in a 48 well plate in triplicate and infected with indicated MOIs of HCMV-eGFP or HSV-1-eGFP. The percentage of herpesvirus-infected cells were monitored in time by subjecting half of the well to flow cytometric analysis (FACSCanto II, BD BioSciences) to detect expression of the eGFP marker after formaldehyde fixing (1%) of the cells. For each flow cytometric measurement at least 2000 events were counted, although for some samples late in lytic infection this number could not be reached due to massive cell death. Flow cytometry data were analyzed using FlowJo software.

### Anti-EBV CRISPR experiments

EBV miRNA sensor experiments were performed in latent EBV-positive SNU719 cells. Cells were transduced with control or anti-EBV miRNA gRNAs (see S1 Table for sequences), selected by puromycin for 2 days, and allowed to recover for ±12 days. Subsequently, cells were transduced with mCherry EBV miRNA sensors for BART5-5p, BART6-3p, or BART16-5p and analyzed by flow cytometry 4 dpi to assess miRNA activity in these cells (FACSCantoII, BD). CRISPR-induced editing of the EBV genome was assessed in anti-BART5 and anti-BART16 gRNAs expressing cells by amplifying these loci by PCR amplification using primers 5’-CGGGCTATATGTCGCCTTAC-3’ and 5’-AGAGGGTGGTGATCTTGGTG-3’ for EBV BART5 and 5’-CCAGGTCAGTGGTTTTGTTTC-3’ and 5’-TGGACCAACCTTAAAGTACCAAC-3’ for EBV BART16. Subsequently, PCR products were cloned in the pCR2.1-TOPO vector according manufacturers’ recommendations (Life technologies), transformed in Turbo competent cells (New England Biolabs) and several clones were mini-prepped (Fermentas) and subjected to sequencing (Macrogen Inc) using the M13-reverse primer 5’-GGAAACAGCTATGACCATG-3’. Sequence analysis was carried out using the Lasergene DNASTAR software package.

To assess the effect of anti-EBV CRISPRs on the EBV latent genome, Akata-Bx1 cells were transduced in triplicate with indicated anti-EBV or control gRNAs expressed from the pSicoR-CRISPR-PuroR vector. Cells were selected by puromycin for 2 days and allowed to recover. The percentage of eGFP-expressing cells as marker for latent EBV infection was monitored by flow cytometry (FACSCanto II, BD) around ±21 days post transduction and analyzed using FlowJo software. For double gRNAs, cells were initially transduced and puromycin-selected with gRNAs expressed from the pSicoR-CRISPR-PuroR vector, and subsequently transduced and blasticidin-selected with gRNAs expressed from the pSicoR-CRISPR-BlastR vector. Double anti-EBV gRNAs were monitored for eGFP expression at ±21 days post infection with the second gRNA.

### gRNA off-target analysis

To study the frequency of off-target editing in the human genome, we assessed the potential occurrence of off-target sites for nine gRNAs via the CRISPR design tool of the Zhang lab (http://crispr.mit.edu/). For each gRNA, we selected the top three scoring potential off-target sites and designed oligos using the primer3web (http://bioinfo.ut.ee/primer3/) to allow amplification of these sites (see S2 Table). We subsequently amplified these sites from gRNA-expressing (cells were transduced with gRNAs at least two weeks prior genomic DNA isolation) and control cells using Platinum Taq DNA polymerase (Life Technologies), purified the samples using the GeneJet PCR purification kit (Thermo Scientific), and assessed the concentration of DNA by the Quant-iT PicoGreen dsDNA Assay kit (Invitrogen) or the Qubit dsDNA BR Assay (Life Technologies). PCR amplicons were pooled and sequenced via 454 sequencing. To allow discrimination between amplicons from control and CRISPR-expressing cells, we designed two primer-sets by adding a 454 linker and a unique barcode to the 5’ end of the primers. Reverse and forward primers for CRISPR-expressing cells contained a 5’-CGTATCGCCTCCCTCGCGCCATCAG **AGACGCACTC**-3’ and 5’-CTATGCGCCTTGCCAGCCCGCTCAG **AGACGCACTC**-3’ addition respectively. Reverse and forward primers for control cells contained 5’-CGTATCGCCTCCCTCGCGCCATCAG **AGACGCACTC**-3’ and 5’-CTATGCGCCTTGCCAGCCCGCTCAG **AGACGCACTC**-3’ addition respectively. Underlined sequences represent 454 adapter sequences, and barcodes are indicated in bold. Amplicons from CRISPR-expressing cells and control cells were mixed in a 2:1 ratio and amplified on beads using the GS Junior Titanium emPCR Kit (Lib-A, Roche) and the GS Junior Titanium Sequencing kit (Roche). 454 sequencing was subsequently performed on a GS Junior System (Roche) and sequences were analyzed by using GS Amplicon Variant Analyzer software (Roche) and/or Seqman Pro software (DNAstar, Lasergene).

### Sequencing of HCMV escape mutants

Supernatants of HSV-1-eGFP infected (MOI 0.5) MRC5 cells expressing gRNAs targeting UL57 (gRNA #1) and UL70 (gRNA #4) were harvested after 21 dpi. Supernatants from gRNA expressing cells containing anti-UL7 and anti-US11 gRNAs were isolated at 2 dpi since these cultures were completely lysed after several days. The genomic target sites of the respective gRNA were amplified by PCR and subjected to 454 sequencing analysis essentially as described in the previous paragraph. The gene-specific regions of primers used for the amplification were (5’-3’): US7-fw: TTTTCCGGTAAACCGAATTG; US7-rev: TCGCTACACGTGTGGAAGAC; US11-fw: CCTCTAACGAGCTCCACAGG; US11-rev: CCGACGTCACTAGATCACCA; UL57-fw: CGCACAGAGACGCCGAAATC; UL57-rev: AATTGCTGGGATCGTTGCGG; UL70-fw: ATGGTGCTGTACTGGCCCTC; and UL70-rev: GTGAACAACGAAACGCTGCAG. Only variants with insertions and deletions at the target sites were used for analysis in Fig 4A, as 454 sequencing cannot accurately assess single nucleotide substitutions.

### qPCR analysis

Real Time quantitative Polymerase Chain Reaction (RT-qPCR) was performed using TaqMan universal PCR master mix (Applied Biossystems, Life Technologies) to quantify the relative amount of HSV-1 genomic DNA present in the supernatants from HSV-1 infected cells and EBV genomic DNA present in latently infected Akata-Bx1 cells. For HSV-1 qPCRs, supernatants were heat inactivated and subjected to triplicate TaqMan measurements using forward primer 5’-TTCTCGTTCCTCACTGCCTCCC-3’, reverse primer 5’-GCAGGCACACGTAACGCACGCT-3’, and FAM-TAMRA HSV-1 specific probe 5’-CGTCTGGACCAACCGCCACAC-3’. For EBV qPCR, genomic DNA was isolated using the Wizard genomic DNA isolation kit (Promega), which was subjected to qPCR using forward primer 5’-GGAACCTGGTCATCCTTTGC-3’, reverse primer 5’-ACGTGCATGGACCGGTTAAT-3’, and FAM-TAMRA TaqMan probe 5’-CGCAGGCACTCGTACTGCTCGCT-3’. Virus-specific TaqMan qPCRs were normalized to RNAse P via the commercial TaqMan Gene Expression Assay according to the instructions of the manufacturer (Applied Biossystems, Life Technologies). The relative viral DNA amount was calculated according to the comparative Ct method (User Bulletin number 2, ABI Prism 7700 Sequence Detection System, P/N 4303859). Relative genome content values for each condition were averaged from triplicate technical measurements and standard deviations were calculated from biological triplicate measurement of the same condition.

To determine the amount of HSV-1 genomes present in latent MRC5-Cas9 cells, genomic DNA was isolated using the Quick-gDNA Miniprep kit (Zymo Research, USA) from gRNA expressing latent cells pre-reactivation (see HSV-1 Latency generation) and subjected to qPCR essentially as described above. For each independently transduced HSV-1 latent MRC5-Cas9 sample the qPCR was performed in technical triplicate. Error bars and average Ct values were determined by calculating the standard deviation across all Ct values from each anti-HSV-1 gRNA biological replicates. All HSV-1 values were normalized to RNAseP levels. The ‘empty vector’ condition was normalized to 1.0.

### HSV-1 plaque assay

HSV-1 plaque assays were performed in triplicate for each biological sample. 75,000 Vero cells were seeded in a 24 well plate well in complete DMEM and infected the following day with various dilutions of supernatant harvested from HSV-1 infected CRISPR-expressing Vero or MRC5 cells. After 3 hours incubation, cells were overlaid with 0.5% agarose (Seakem LE Agarose, Lonza AG, Switzerland) solution in complete DMEM and cultured for 3 days to allow the formation of plaques to occur. Afterwards, cells were fixed overnight at room temperature with 1% formaldehyde and stained using 0.5% crystal violet solution. After 3 washes with water, plates were allowed to dry and the numbers of plaques were counted by eye. Virus titers were calculated as plaque-forming units/ml (pfu/ml). If for a condition in the lowest dilution no plaques were detected, this was scored as not detected (ND). Images of plaques were taken using the EVOS FL Cell Imager (Life Technologies).

### HSV-1 quiescency model

The HSV-2 quiescency model from Russell *et al*. [47, 48] was adapted for HSV-1 in MRC5 cells. In short, MRC5-Cas9 cells were seeded in multiple 6-wells plates 3 days prior infection at 210.000 cells/well. After 3 days, the cells were infected with 105k viral HSV-1-eGFP particles/well at 37°C for one hour. The infected MRC5-Cas9 cells were subsequently washed twice with complete medium and incubated in a CO_2_ incubator at 42°C for 4 days. The culture medium was replaced every day. After 4 days, the cells were returned to regular cell culture conditions (37°C 5% CO2). Next, cells were monitored by fluorescence microscopy for 6 days to exclude wells displaying signs of spontaneously reactivation, which occurred in ± half of the wells. Cells were subsequently harvested and plated at 150,000 cells/well in 12-wells plates. The next day, wells not displaying signs of spontaneous reactivation were transduced with gRNA-carrying lentivirus and selected for after 3 days using puromycin (2μg/ml) for the duration of 3 days. MRC5-Cas9 cells containing quiescent HSV-1-eGFP were counted and 40,000 cells/well (48-well plate) were seeded in triplicate in 48 well plates. The remainder was pelleted and subjected to gDNA isolation using the Quick-gDNA Miniprep kit (Zymo Research, USA). The gDNA was subjected to qPCR to determine the relative HSV-1 genome content. Quiescent MRC5-Cas9 cells were infected the next day with AD169 HCMV (ATCC VR538) to trigger HSV-1-eGFP reactivation. Three days later, cells were harvested, fixed with formaldehyde (1%), and measured by flow cytometry to assess the percentage of eGFP-positive cells as a measure for cells with replicating HSV-1-eGFP.

### Analysis of CRISPR/Cas9-mediated genome editing during HSV-1 latently

gRNA target sites were amplified from gRNA-expressing quiescent MRC5 cells using the following gene-specific primers: UL8-fw 5’-TAGAAATCCCGCAGCTCCGTC-3’, UL8-rev 5’-GGGGCGGTGAACTTTAGCAC-3’, UL29-fw 5’-GAGGGCGTCAGTTTCAGGGAC-3’, UL29-rev 5’-GATTCATTCCCCAACCCCGGTC-3’, UL52-fw 5’-GCGCGGATCATCTCATATTGTTCC-3’ and UL52–rev 5’- GACGAACATGGGTCGGGTTC-3’. Derived amplicons were isolated from 2% agarose gels by gel extraction (GeneJET PCR Purification Kit, ThermoFisher Scientific) and subjected to a second PCR reaction to increase product yield and add sequence tags (lower case sequence) for subsequent barcoding and Illumina adapter introduction: UL8_flank_fw 5’-tcgtcggcagcgtcagatgtgtataagagacagGGCGTTGCGACATACAAAATAC-3’, UL8_flank_rev 5’-gtctcgtgggctcggagatgtgtataagagacagTATAAGTCTCGGGACCGCACTC-3’, UL29_flank_fw 5’-tcgtcggcagcgtcagatgtgtataagagacagGTGCGAGAACCCACGACCAC-3’, UL29_flank_rev 5’- gtctcgtgggctcggagatgtgtataagagacagCTCGGGAGACATACCTTGTCG-3’, UL52_flank_fw -tcgtcggcagcgtcagatgtgtataagagacagTCTCATATTGTTCCTCGGGGCG-3’ and UL52_flank_rev 5’-gtctcgtgggctcggagatgtgtataagagacagTCTTCGAACCTGTCTTGCTCCG-3’. Expand High Fidelity PCR system (Roche) was used for all PCR reactions according to manufacturer’s instructions.

Amplified DNA was isolated from 2% agarose gels (GeneJET PCR Purification Kit, ThermoFisher Scientific), barcoded and prepared for Illumina MiSeq sequencing using the 16S Metagenomic Sequencing Library Preparation kit (Illumina). DNA concentrations were determined by the Qubit fluorometer 2.0 (Life Technologies, USA) with the Qubit dsDNA High Specificity assay kit. DNA libraries were sequenced by Illumina MiSeq (250bp paired-end). Obtained sequences were quality-checked with FASTQC v0.11.3 (http://www.bioinformatics.babraham.ac.uk/projects/fastqc/) and trimmed with seqtk version 1.0-r31. Sequences were aligned to the viral reference sequences by using Bowtie2 version 2.2.6 [90] using the sensitive-local alignment mode. Alignments were converted to bam format and indexed with samtools version 1.3 [91] and analyzed in Tablet version 1.14.04.10 [92] for CRISPR/Cas9 specific indels at the gRNA target site.

## Supporting Information

S1 Fig EBV BART miRNAs are active in SNU719 cells and can be targeted by CRISPR/Cas9. **a)** Schematic representation of miRNA sensor constructs used to asses specific EBV miRNA activity in EBV-positive cells. The human EF1a promoter drives expression of the fluorescent mCherry gene which holds a single perfect target site for a given EBV miRNA in its 3’UTR. **b)** EBV-positive SNU719 cells were transduced with the indicated EBV miRNA sensor constructs or vector control lacking miRNA targetsites. The mCherry-reporter expression was assessed by flow cytometry and showed a clear downregulation caused by silencing activity of the indicated EBV BART miRNA **c)** Schematic representation of the studies presented Fig 1A. Briefly, EBV-positive SNU719 cells are transduced with a given miRNA sensor construct (lower panels) or control vector (top panels). The control vector induces high level of mCherry expression, whereas the miRNA reporter vector display reduced mCherry levels as the given EBV miRNA is expressed in the EBV+ cells thereby repressing reporter expression (see b)). Subsequently, anti-miRNA gRNAs are introduced that induce site-specific mutations at the miRNA target genes. Therefore, miRNA-induced silencing of the miRNA reporter is abrogated resulting in enhanced expression of mCherry.
(EPS)

S2 Fig No signs for off-target activity of anti-HSV-1 gRNAs. To assess the activity of the three most potent anti-HSV-1 gRNAs towards potential off-target sites within the human genome, the top three predicted off-target sites (as assessed by the CRISPR design tool crispr.mit.edu) were PCR amplified from gRNA-expressing and controls cells. These were subsequently subjected to standard Sanger sequencing. CRISPR/Cas9-mediated genome-editing normally results in the emergence of a mixed read in the sequencing histogram initiating 3 basepairs upstream the PAM sequence (the Cas9 cleavage site). Since there are no apparent mixed reads visible at the Cas9 cleavage sites, it can be concluded that no overt off-target editing has occurred at these sites. Similar conclusions can be drawn from deep-sequencing analysis of 18 additional off-target sites for six gRNAs targeting HCMV and EBV (S3 Table).
(EPS)

S3 Fig HSV-1 quiescency model in MRC5 cells. The HSV-2 quiescency model as described by Russell and Preston [47, 48] was adapted for MRC5 cells as described in the methods section. **a)** Displayed are MRC5 cells harboring quiescent HSV-1 as visualized by light microscopy before (left panels) and 3 days after reactivation with HCMV (right panels). **b)** Cells were analyzed by flow cytometry to assess eGFP levels as measure for reactivated and replicating HSV-1.
(EPS)

S4 Fig CRISPR/Cas9-mediated HSV-1 editing during quiescence. Sequencing of CRISPR-targeted quiescent HSV-1 genomes show low frequency editing at the indicated target sites. The HSV-1 genomic loci of UL8 and UL52 were amplified by PCR and subjected to Illumina sequencing. The gRNA-target sites are displayed in bold, the PAM sequence as red, underlined text, and the cleavage site as a triangle. Red nucleotides correspond to substitutions that do not match the reference sequence. The number of times each variant has been sequenced and the number of deleted/inserted nucleotides is indicated. Control vector treated quiescent MRC5 cells did not show any indels at these specific sites.
(EPS)

S1 Table gRNA sequences used in this study. (PDF)

S2 Table Primer sequences for off-target analysis. (PDF)

S3 Table No signs for off-target activity of anti-HCMV and anti-EBV gRNAs. (PDF)

## References

[1] Pellett PE, Roizman B. Herpesviridae In: Knipe D, Howley P, editors. Fields Virology 2 6 ed: Lippincott, Williams & Wilkins; 2013 p. 1802–22.

[2] Liesegang TJ, Melton LJ 3rd, Daly PJ, Ilstrup DM. Epidemiology of ocular herpes simplex. Incidence in Rochester, Minn, 1950 through 1982. Archives of ophthalmology. 1989;107(8):1155–9. Epub 1989/08/01. . 2787981 10.1001/archopht.1989.01070020221029

[3] Hill GM, Ku ES, Dwarakanathan S. Herpes simplex keratitis. Disease-a-month: DM. 2014;60(6):239–46. Epub 2014/06/08. 10.1016/j.disamonth.2014.03.003 . 24906668

[4] Arvin AM, Gilden D. Varicella-Zoster Virus In: Knipe D, Howley P, editors. Fields Virology. 2 6 ed: Lippincott Wiliams & Wilkins; 2013 p. 2015–57.

[5] Mocarski ES, Shenk T, Griffiths PD, F. PR. Cytomegaloviruses In: Knipe D, Howley PM, editors. Fields Virology 2 6 ed: Lippincott, Williams & Wilkins; 2013 p. 1960–2014.

[6] Vora SB, Englund JA. Cytomegalovirus in immunocompromised children. Current opinion in infectious diseases. 2015;28(4):323–9. Epub 2015/06/23. 10.1097/QCO.0000000000000174 . 26098503

[7] Longnecker RM, Kieff E, Cohen JI. Epstein-Barr Virus In: Knipe D, Howley PM, editors. Fields Virology 2 6 ed: Lippincott, Williams & Wilkins; 2013 p. 1898–959.

[8] Damania BA, Cesarman E. Kaposi's Sarcoma-Associated Herpesvirus In: Knipe D, Howley P, editors. Fields Virology. 2 6 ed: Lippincott Wiliams & Wilkins; 2013 p. 2080–128.

[9] Piret J, Boivin G. Antiviral drug resistance in herpesviruses other than cytomegalovirus. Reviews in medical virology. 2014;24(3):186–218. Epub 2014/03/08. 10.1002/rmv.1787 . 24604770

[10] Andrei G, De Clercq E, Snoeck R. Viral DNA Polymerase Inhibitors In: Raney KD, Gotte M, Cameron CE, editors. Viral Genome Replication: Springer US; 2009 p. 481–526.

[11] Price AM, Luftig MA. Dynamic Epstein-Barr virus gene expression on the path to B-cell transformation. Advances in virus research. 2014;88:279–313. Epub 2014/01/01. 10.1016/B978-0-12-800098-4.00006–4 . 24373315 PMC4911173

[12] Mali P, Esvelt KM, Church GM. Cas9 as a versatile tool for engineering biology. Nature methods. 2013;10(10):957–63. Epub 2013/10/01. 10.1038/nmeth.2649 . 24076990 PMC4051438

[13] Horvath P, Barrangou R. CRISPR/Cas, the immune system of bacteria and archaea. Science. 2010;327(5962):167–70. Epub 2010/01/09. 10.1126/science.1179555 . 20056882

[14] Wiedenheft B, Sternberg SH, Doudna JA. RNA-guided genetic silencing systems in bacteria and archaea. Nature. 2012;482(7385):331–8. Epub 2012/02/18. 10.1038/nature10886 . 22337052

[15] Hsu PD, Lander ES, Zhang F. Development and applications of CRISPR-Cas9 for genome engineering. Cell. 2014;157(6):1262–78. Epub 2014/06/07. 10.1016/j.cell.2014.05.010 . 24906146 PMC4343198

[16] Sternberg SH, Doudna JA. Expanding the Biologist's Toolkit with CRISPR-Cas9. Molecular cell. 2015;58(4):568–74. Epub 2015/05/23. 10.1016/j.molcel.2015.02.032 . 26000842

[17] Cong L, Ran FA, Cox D, Lin S, Barretto R, Habib N, et al Multiplex genome engineering using CRISPR/Cas systems. Science. 2013;339(6121):819–23. Epub 2013/01/05. 10.1126/science.1231143 23287718 PMC3795411

[18] Mali P, Yang L, Esvelt KM, Aach J, Guell M, DiCarlo JE, et al RNA-guided human genome engineering via Cas9. Science. 2013;339(6121):823–6. Epub 2013/01/05. 10.1126/science.1232033 23287722 PMC3712628

[19] Mandal PK, Ferreira LM, Collins R, Meissner TB, Boutwell CL, Friesen M, et al Efficient ablation of genes in human hematopoietic stem and effector cells using CRISPR/Cas9. Cell stem cell. 2014;15(5):643–52. Epub 2014/12/18. 10.1016/j.stem.2014.10.004 25517468 PMC4269831

[20] Wang H, Yang H, Shivalila CS, Dawlaty MM, Cheng AW, Zhang F, et al One-step generation of mice carrying mutations in multiple genes by CRISPR/Cas-mediated genome engineering. Cell. 2013;153(4):910–8. Epub 2013/05/07. 10.1016/j.cell.2013.04.025 . 23643243 PMC3969854

[21] Yang H, Wang H, Shivalila CS, Cheng AW, Shi L, Jaenisch R. One-Step Generation of Mice Carrying Reporter and Conditional Alleles by CRISPR/Cas-Mediated Genome Engineering. Cell. 2013;154(6):1370–9. Epub 2013/09/03. 10.1016/j.cell.2013.08.022 . 23992847 PMC3961003

[22] Li D, Qiu Z, Shao Y, Chen Y, Guan Y, Liu M, et al Heritable gene targeting in the mouse and rat using a CRISPR-Cas system. Nature biotechnology. 2013;31(8):681–3. Epub 2013/08/10. 10.1038/nbt.2661 . 23929336

[23] Bi Y, Sun L, Gao D, Ding C, Li Z, Li Y, et al High-efficiency targeted editing of large viral genomes by RNA-guided nucleases. PLoS pathogens. 2014;10(5):e1004090 Epub 2014/05/03. 10.1371/journal.ppat.1004090 24788700 PMC4006927

[24] Kennedy EM, Kornepati AV, Goldstein M, Bogerd HP, Poling BC, Whisnant AW, et al Inactivation of the human papillomavirus E6 or E7 gene in cervical carcinoma cells by using a bacterial CRISPR/Cas RNA-guided endonuclease. Journal of virology. 2014;88(20):11965–72. Epub 2014/08/08. 10.1128/jvi.01879-14 ; PubMed Central PMCID: PMCPmc4178730. 25100830 PMC4178730

[25] Zhen S, Hua L, Takahashi Y, Narita S, Liu YH, Li Y. In vitro and in vivo growth suppression of human papillomavirus 16-positive cervical cancer cells by CRISPR/Cas9. Biochemical and biophysical research communications. 2014;450(4):1422–6. Epub 2014/07/22. 10.1016/j.bbrc.2014.07.014 . 25044113

[26] Kennedy EM, Bassit LC, Mueller H, Kornepati AV, Bogerd HP, Nie T, et al Suppression of hepatitis B virus DNA accumulation in chronically infected cells using a bacterial CRISPR/Cas RNA-guided DNA endonuclease. Virology. 2014;476c:196–205. Epub 2015/01/02. 10.1016/j.virol.2014.12.001 . 25553515 PMC4323668

[27] Russell TA, Stefanovic T, Tscharke DC. Engineering herpes simplex viruses by infection-transfection methods including recombination site targeting by CRISPR/Cas9 nucleases. Journal of virological methods. 2015;213:18–25. Epub 2014/12/06. 10.1016/j.jviromet.2014.11.009 . 25479355

[28] Suenaga T, Kohyama M, Hirayasu K, Arase H. Engineering large viral DNA genomes using the CRISPR-Cas9 system. Microbiology and immunology. 2014;58(9):513–22. Epub 2014/07/22. 10.1111/1348-0421.12180 . 25040500 PMC7168497

[29] Wang J, Quake SR. RNA-guided endonuclease provides a therapeutic strategy to cure latent herpesviridae infection. Proceedings of the National Academy of Sciences of the United States of America. 2014;111(36):13157–62. Epub 2014/08/27. 10.1073/pnas.1410785111 . 25157128 PMC4246930

[30] Wollebo HS, Bellizzi A, Kaminski R, Hu W, White MK, Khalili K. CRISPR/Cas9 System as an Agent for Eliminating Polyomavirus JC Infection. PloS one. 2015;10(9):e0136046 Epub 2015/09/12. 10.1371/journal.pone.0136046 26360417 PMC4567079

[31] Price AA, Sampson TR, Ratner HK, Grakoui A, Weiss DS. Cas9-mediated targeting of viral RNA in eukaryotic cells. Proceedings of the National Academy of Sciences of the United States of America. 2015;112(19):6164–9. Epub 2015/04/29. 10.1073/pnas.1422340112 25918406 PMC4434742

[32] Ebina H, Misawa N, Kanemura Y, Koyanagi Y. Harnessing the CRISPR/Cas9 system to disrupt latent HIV-1 provirus. Scientific reports. 2013;3:2510 Epub 2013/08/27. 10.1038/srep02510 ; PubMed Central PMCID: PMCPmc3752613. 23974631 PMC3752613

[33] Hu W, Kaminski R, Yang F, Zhang Y, Cosentino L, Li F, et al RNA-directed gene editing specifically eradicates latent and prevents new HIV-1 infection. Proceedings of the National Academy of Sciences of the United States of America. 2014;111(31):11461–6. Epub 2014/07/23. 10.1073/pnas.1405186111 ; PubMed Central PMCID: PMCPmc4128125. 25049410 PMC4128125

[34] Liao HK, Gu Y, Diaz A, Marlett J, Takahashi Y, Li M, et al Use of the CRISPR/Cas9 system as an intracellular defense against HIV-1 infection in human cells. Nature communications. 2015;6:6413 Epub 2015/03/11. 10.1038/ncomms7413 . 25752527

[35] Zhang Y, Yin C, Zhang T, Li F, Yang W, Kaminski R, et al CRISPR/gRNA-directed synergistic activation mediator (SAM) induces specific, persistent and robust reactivation of the HIV-1 latent reservoirs. Scientific reports. 2015;5:16277 Epub 2015/11/06. 10.1038/srep16277 26538064 PMC4633726

[36] Kaminski R, Chen Y, Fischer T, Tedaldi E, Napoli A, Zhang Y, et al Elimination of HIV-1 Genomes from Human T-lymphoid Cells by CRISPR/Cas9 Gene Editing. Scientific reports. 2016;6:22555 10.1038/srep22555 26939770 PMC4778041

[37] Reisman D, Yates J, Sugden B. A putative origin of replication of plasmids derived from Epstein-Barr virus is composed of two cis-acting components. Molecular and cellular biology. 1985;5(8):1822–32. Epub 1985/08/01. ; PubMed Central PMCID: PMCPmc366897. 3018528 10.1128/mcb.5.8.1822-1832.1985 PMC366897

[38] Rawlins DR, Milman G, Hayward SD, Hayward GS. Sequence-specific DNA binding of the Epstein-Barr virus nuclear antigen (EBNA-1) to clustered sites in the plasmid maintenance region. Cell. 1985;42(3):859–68. Epub 1985/10/01. . 2996781 10.1016/0092-8674(85)90282-x

[39] Molesworth SJ, Lake CM, Borza CM, Turk SM, Hutt-Fletcher LM. Epstein-Barr virus gH is essential for penetration of B cells but also plays a role in attachment of virus to epithelial cells. Journal of virology. 2000;74(14):6324–32. Epub 2000/06/23. ; PubMed Central PMCID: PMCPmc112138. 10864642 10.1128/jvi.74.14.6324-6332.2000 PMC112138

[40] Yu D, Silva MC, Shenk T. Functional map of human cytomegalovirus AD169 defined by global mutational analysis. Proceedings of the National Academy of Sciences of the United States of America. 2003;100(21):12396–401. Epub 2003/10/02. 10.1073/pnas.1635160100 ; PubMed Central PMCID: PMCPmc218769. 14519856 PMC218769

[41] Dunn W, Chou C, Li H, Hai R, Patterson D, Stolc V, et al Functional profiling of a human cytomegalovirus genome. Proceedings of the National Academy of Sciences of the United States of America. 2003;100(24):14223–8. Epub 2003/11/19. 10.1073/pnas.2334032100 ; PubMed Central PMCID: PMCPmc283573. 14623981 PMC283573

[42] Sampaio KL, Cavignac Y, Stierhof YD, Sinzger C. Human cytomegalovirus labeled with green fluorescent protein for live analysis of intracellular particle movements. Journal of virology. 2005;79(5):2754–67. Epub 2005/02/15. 10.1128/jvi.79.5.2754–2767.2005 ; PubMed Central PMCID: PMCPmc548422. 15708994 PMC548422

[43] Spector DJ, Yetming K. UL84-independent replication of human cytomegalovirus strain TB40/E. Virology. 2010;407(2):171–7. Epub 2010/09/22. 10.1016/j.virol.2010.08.029 . 20855098

[44] McGeoch DJ, Rixon FJ, Davison AJ. Topics in herpesvirus genomics and evolution. Virus research. 2006;117(1):90–104. Epub 2006/02/24. 10.1016/j.virusres.2006.01.002 . 16490275

[45] Crute JJ, Mocarski ES, Lehman IR. A DNA helicase induced by herpes simplex virus type 1. Nucleic acids research. 1988;16(14A):6585–96. Epub 1988/07/25. 2840645 10.1093/nar/16.14.6585 PMC338315

[46] Crute JJ, Tsurumi T, Zhu LA, Weller SK, Olivo PD, Challberg MD, et al Herpes simplex virus 1 helicase-primase: a complex of three herpes-encoded gene products. Proceedings of the National Academy of Sciences of the United States of America. 1989;86(7):2186–9. Epub 1989/04/01. 2538835 10.1073/pnas.86.7.2186 PMC286876

[47] Russell J, Preston CM. An in vitro latency system for herpes simplex virus type 2. The Journal of general virology. 1986;67 (Pt 2):397–403. Epub 1986/02/01. 10.1099/0022-1317-67-2-397 . 3003243

[48] Russell J, Stow ND, Stow EC, Preston CM. Herpes simplex virus genes involved in latency in vitro. The Journal of general virology. 1987;68 (Pt 12):3009–18. Epub 1987/12/01. 10.1099/0022-1317-68-12-3009 . 2826646

[49] Feng WH, Hong G, Delecluse HJ, Kenney SC. Lytic induction therapy for Epstein-Barr virus-positive B-cell lymphomas. Journal of virology. 2004;78(4):1893–902. Epub 2004/01/30. ; PubMed Central PMCID: PMCPmc369434. 14747554 10.1128/JVI.78.4.1893-1902.2004 PMC369434

[50] Elbadawy HM, Gailledrat M, Desseaux C, Salvalaio G, Di Iorio E, Ferrari B, et al Gene transfer of integration defective anti-HSV-1 meganuclease to human corneas ex vivo. Gene therapy. 2014;21(3):272–81. Epub 2014/01/17. 10.1038/gt.2013.82 . 24430237

[51] Aubert M, Boyle NM, Stone D, Stensland L, Huang ML, Magaret AS, et al In vitro Inactivation of Latent HSV by Targeted Mutagenesis Using an HSV-specific Homing Endonuclease. Molecular therapy Nucleic acids. 2014;3:e146 Epub 2014/02/06. 10.1038/mtna.2013.75 ; PubMed Central PMCID: PMCPmc3951911. 24496438 PMC3951911

[52] Reisman D, Sugden B. trans activation of an Epstein-Barr viral transcriptional enhancer by the Epstein-Barr viral nuclear antigen 1. Molecular and cellular biology. 1986;6(11):3838–46. Epub 1986/11/01. ; PubMed Central PMCID: PMCPmc367146. 3025615 10.1128/mcb.6.11.3838-3846.1986 PMC367146

[53] Sugden B, Marsh K, Yates J. A vector that replicates as a plasmid and can be efficiently selected in B-lymphoblasts transformed by Epstein-Barr virus. Molecular and cellular biology. 1985;5(2):410–3. Epub 1985/02/01. ; PubMed Central PMCID: PMCPmc366725. 2983194 10.1128/mcb.5.2.410-413.1985 PMC366725

[54] Yates JL, Warren N, Sugden B. Stable replication of plasmids derived from Epstein-Barr virus in various mammalian cells. Nature. 1985;313(6005):812–5. Epub 1985/02/06. . 2983224 10.1038/313812a0

[55] Yin Q, Flemington EK. siRNAs against the Epstein Barr virus latency replication factor, EBNA1, inhibit its function and growth of EBV-dependent tumor cells. Virology. 2006;346(2):385–93. Epub 2005/12/14. 10.1016/j.virol.2005.11.021 . 16343579

[56] Lee EK, Kim SY, Noh KW, Joo EH, Zhao B, Kieff E, et al Small molecule inhibition of Epstein-Barr virus nuclear antigen-1 DNA binding activity interferes with replication and persistence of the viral genome. Antiviral research. 2014;104:73–83. Epub 2014/02/04. 10.1016/j.antiviral.2014.01.018 ; PubMed Central PMCID: PMCPmc3964181. 24486954 PMC3964181

[57] Kang MS, Lee EK, Soni V, Lewis TA, Koehler AN, Srinivasan V, et al Roscovitine inhibits EBNA1 serine 393 phosphorylation, nuclear localization, transcription, and episome maintenance. Journal of virology. 2011;85(6):2859–68. Epub 2011/01/07. 10.1128/jvi.01628-10 ; PubMed Central PMCID: PMCPmc3067954. 21209116 PMC3067954

[58] Nasimuzzaman M, Kuroda M, Dohno S, Yamamoto T, Iwatsuki K, Matsuzaki S, et al Eradication of Epstein-Barr virus episome and associated inhibition of infected tumor cell growth by adenovirus vector-mediated transduction of dominant-negative EBNA1. Molecular therapy: the journal of the American Society of Gene Therapy. 2005;11(4):578–90. Epub 2005/03/18. 10.1016/j.ymthe.2004.12.017 . 15771960

[59] Kim do N, Seo MK, Choi H, Kim SY, Shin HJ, Yoon AR, et al Characterization of naturally Epstein-Barr virus-infected gastric carcinoma cell line YCCEL1. The Journal of general virology. 2013;94(Pt 3):497–506. Epub 2012/11/24. 10.1099/vir.0.045237–0 . 23175241

[60] Zheng Q, Cai X, Tan MH, Schaffert S, Arnold CP, Gong X, et al Precise gene deletion and replacement using the CRISPR/Cas9 system in human cells. BioTechniques. 2014;57(3):115–24. Epub 2014/09/12. 10.2144/000114196 . 25209046

[61] Yuen KS, Chan CP, Wong NH, Ho CH, Ho TH, Lei T, et al CRISPR/Cas9-mediated genome editing of Epstein-Barr virus in human cells. The Journal of general virology. 2015;96(Pt 3):626–36. Epub 2014/12/17. 10.1099/jgv.0.000012 . 25502645

[62] Riballo E, Kuhne M, Rief N, Doherty A, Smith GC, Recio MJ, et al A pathway of double-strand break rejoining dependent upon ATM, Artemis, and proteins locating to gamma-H2AX foci. Molecular cell. 2004;16(5):715–24. Epub 2004/12/03. 10.1016/j.molcel.2004.10.029 . 15574327

[63] Rajcani J, Durmanova V. Mechanisms of replication of alpha- and betaherpesviruses and their pathogenesis. Bratislavske lekarske listy. 2001;102(11):505–14. Epub 2002/03/21. . 11901707

[64] Doench JG, Hartenian E, Graham DB, Tothova Z, Hegde M, Smith I, et al Rational design of highly active sgRNAs for CRISPR-Cas9-mediated gene inactivation. Nature biotechnology. 2014;32(12):1262–7. Epub 2014/09/04. 10.1038/nbt.3026 25184501 PMC4262738

[65] Kuscu C, Arslan S, Singh R, Thorpe J, Adli M. Genome-wide analysis reveals characteristics of off-target sites bound by the Cas9 endonuclease. Nature biotechnology. 2014;32(7):677–83. Epub 2014/05/20. 10.1038/nbt.2916 . 24837660

[66] Wu X, Scott DA, Kriz AJ, Chiu AC, Hsu PD, Dadon DB, et al Genome-wide binding of the CRISPR endonuclease Cas9 in mammalian cells. Nature biotechnology. 2014;32(7):670–6. Epub 2014/04/23. 10.1038/nbt.2889 24752079 PMC4145672

[67] Pybus OG, Rambaut A. Evolutionary analysis of the dynamics of viral infectious disease. Nature reviews Genetics. 2009;10(8):540–50. Epub 2009/07/01. 10.1038/nrg2583 . 19564871 PMC7097015

[68] Sakaoka H, Kurita K, Iida Y, Takada S, Umene K, Kim YT, et al Quantitative analysis of genomic polymorphism of herpes simplex virus type 1 strains from six countries: studies of molecular evolution and molecular epidemiology of the virus. The Journal of general virology. 1994;75 (Pt 3):513–27. Epub 1994/03/01. . 8126449 10.1099/0022-1317-75-3-513

[69] Fu Y, Foden JA, Khayter C, Maeder ML, Reyon D, Joung JK, et al High-frequency off-target mutagenesis induced by CRISPR-Cas nucleases in human cells. Nature biotechnology. 2013;31(9):822–6. Epub 2013/06/25. 10.1038/nbt.2623 ; PubMed Central PMCID: PMCPmc3773023. 23792628 PMC3773023

[70] Ran FA, Hsu PD, Lin CY, Gootenberg JS, Konermann S, Trevino AE, et al Double nicking by RNA-guided CRISPR Cas9 for enhanced genome editing specificity. Cell. 2013;154(6):1380–9. Epub 2013/09/03. 10.1016/j.cell.2013.08.021 23992846 PMC3856256

[71] Fu Y, Sander JD, Reyon D, Cascio VM, Joung JK. Improving CRISPR-Cas nuclease specificity using truncated guide RNAs. Nature biotechnology. 2014;32(3):279–84. Epub 2014/01/28. 10.1038/nbt.2808 ; PubMed Central PMCID: PMCPmc3988262. 24463574 PMC3988262

[72] Kleinstiver BP, Pattanayak V, Prew MS, Tsai SQ, Nguyen NT, Zheng Z, et al High-fidelity CRISPR-Cas9 nucleases with no detectable genome-wide off-target effects. Nature. 2016;529(7587):490–5. Epub 2016/01/07. 10.1038/nature16526 . 26735016 PMC4851738

[73] Kleinstiver BP, Prew MS, Tsai SQ, Topkar VV, Nguyen NT, Zheng Z, et al Engineered CRISPR-Cas9 nucleases with altered PAM specificities. Nature. 2015;523(7561):481–5. Epub 2015/06/23. 10.1038/nature14592 26098369 PMC4540238

[74] Slaymaker IM, Gao L, Zetsche B, Scott DA, Yan WX, Zhang F. Rationally engineered Cas9 nucleases with improved specificity. Science. 2016;351(6268):84–8. Epub 2015/12/03. 10.1126/science.aad5227 26628643 PMC4714946

[75] Nicoll MP, Proenca JT, Efstathiou S. The molecular basis of herpes simplex virus latency. FEMS microbiology reviews. 2012;36(3):684–705. Epub 2011/12/14. 10.1111/j.1574-6976.2011.00320.x 22150699 PMC3492847

[76] Coleman HM, Connor V, Cheng ZS, Grey F, Preston CM, Efstathiou S. Histone modifications associated with herpes simplex virus type 1 genomes during quiescence and following ICP0-mediated de-repression. The Journal of general virology. 2008;89(Pt 1):68–77. Epub 2007/12/20. 10.1099/vir.0.83272–0 18089730 PMC2884978

[77] Ferenczy MW, Ranayhossaini DJ, Deluca NA. Activities of ICP0 involved in the reversal of silencing of quiescent herpes simplex virus 1. Journal of virology. 2011;85(10):4993–5002. Epub 2011/03/18. 10.1128/JVI.02265-10 21411540 PMC3126212

[78] Wang D, Gao G. State-of-the-art human gene therapy: part II. Gene therapy strategies and clinical applications. Discovery medicine. 2014;18(98):151–61. Epub 2014/09/18. . 25227756 PMC4440458

[79] Flotte TR. Birth of a new therapeutic platform: 47 years of adeno-associated virus biology from virus discovery to licensed gene therapy. Molecular therapy: the journal of the American Society of Gene Therapy. 2013;21(11):1976–81. Epub 2013/11/10. 10.1038/mt.2013.226 ; PubMed Central PMCID: PMCPmc3831048. 24201212 PMC3831048

[80] Wang D, Gao G. State-of-the-art human gene therapy: part I. Gene delivery technologies. Discovery medicine. 2014;18(97):67–77. Epub 2014/08/06. . 25091489 PMC4440413

[81] Bartlett JS, Samulski RJ, McCown TJ. Selective and rapid uptake of adeno-associated virus type 2 in brain. Human gene therapy. 1998;9(8):1181–6. Epub 1998/06/13. 10.1089/hum.1998.9.8–1181 . 9625257

[82] D'Astolfo DS, Pagliero RJ, Pras A, Karthaus WR, Clevers H, Prasad V, et al Efficient intracellular delivery of native proteins. Cell. 2015;161(3):674–90. Epub 2015/04/25. 10.1016/j.cell.2015.03.028 . 25910214

[83] Zuris JA, Thompson DB, Shu Y, Guilinger JP, Bessen JL, Hu JH, et al Cationic lipid-mediated delivery of proteins enables efficient protein-based genome editing in vitro and in vivo. Nature biotechnology. 2015;33(1):73–80. Epub 2014/10/31. 10.1038/nbt.3081 25357182 PMC4289409

[84] Terhune S, Torigoi E, Moorman N, Silva M, Qian Z, Shenk T, et al Human cytomegalovirus UL38 protein blocks apoptosis. Journal of virology. 2007;81(7):3109–23. Epub 2007/01/05. 10.1128/jvi.02124-06 ; PubMed Central PMCID: PMCPmc1866066. 17202209 PMC1866066

[85] La Boissiere S, Izeta A, Malcomber S, O'Hare P. Compartmentalization of VP16 in cells infected with recombinant herpes simplex virus expressing VP16-green fluorescent protein fusion proteins. Journal of virology. 2004;78(15):8002–14. Epub 2004/07/16. 10.1128/JVI.78.15.8002–8014.2004 15254172 PMC446094

[86] Ventura A, Meissner A, Dillon CP, McManus M, Sharp PA, Van Parijs L, et al Cre-lox-regulated conditional RNA interference from transgenes. Proceedings of the National Academy of Sciences of the United States of America. 2004;101(28):10380–5. Epub 2004/07/09. 10.1073/pnas.0403954101 ; PubMed Central PMCID: PMCPmc478580. 15240889 PMC478580

[87] Osborn MJ, Panoskaltsis-Mortari A, McElmurry RT, Bell SK, Vignali DA, Ryan MD, et al A picornaviral 2A-like sequence-based tricistronic vector allowing for high-level therapeutic gene expression coupled to a dual-reporter system. Molecular therapy: the journal of the American Society of Gene Therapy. 2005;12(3):569–74. Epub 2005/06/21. 10.1016/j.ymthe.2005.04.013 . 15964244

[88] Jinek M, Chylinski K, Fonfara I, Hauer M, Doudna JA, Charpentier E. A programmable dual-RNA-guided DNA endonuclease in adaptive bacterial immunity. Science. 2012;337(6096):816–21. Epub 2012/06/30. 10.1126/science.1225829 . 22745249 PMC6286148

[89] van de Weijer ML, Bassik MC, Luteijn RD, Voorburg CM, Lohuis MA, Kremmer E, et al A high-coverage shRNA screen identifies TMEM129 as an E3 ligase involved in ER-associated protein degradation. Nature communications. 2014;5:3832 Epub 2014/05/09. 10.1038/ncomms4832 ; PubMed Central PMCID: PMCPmc4024746. 24807418 PMC4024746

[90] Langmead B, Salzberg SL. Fast gapped-read alignment with Bowtie 2. Nature methods. 2012;9(4):357–9. Epub 2012/03/06. 10.1038/nmeth.1923 22388286 PMC3322381

[91] Li H, Handsaker B, Wysoker A, Fennell T, Ruan J, Homer N, et al The Sequence Alignment/Map format and SAMtools. Bioinformatics. 2009;25(16):2078–9. Epub 2009/06/10. 10.1093/bioinformatics/btp352 19505943 PMC2723002

[92] Milne I, Stephen G, Bayer M, Cock PJA, Pritchard L, Cardle L, et al Using Tablet for visual exploration of second-generation sequencing data. Brief Bioinform. 2013;14(2):193–202. 10.1093/bib/bbs012 . 22445902

