# Supplementary material for: CRISPR/Cas9-Mediated Genome Editing of Herpesviruses Limits Productive and Latent Infections
Source: PLoS Pathog. 2016 Jun 30;12(6):e1005701. doi: 10.1371/journal.ppat.1005701 (PMC4928872; doi:10.1371/journal.ppat.1005701)
Supplement: S1 Table — (PDF) [file ppat.1005701.s005.pdf]

S1 Table. gRNA sequences used in this study

Target gene/region	Genome	CRISPR gRNA sequence[a]	Used for double gRNA experiments
HLA-A	Human	GAAGGAGACGCTGCAGCGCA	
B2M	Human	GAGTAGCGCGAGCACAGCTA	
eGFP	-	GGAGCGCACCATCTTCTTCA	
BART5 miRNA	EBV	GGGCAGCTATATTCACCTTG	
BART16 miRNA	EBV	GGTGTGGAATTTAGATAGAG	
BART6 miRNA	EBV	GTGACCTTGTTGGTACTTTA	
dyad #1	EBV	GATAGCATATGCTTCCCGTT	
dyad #2	EBV	GTAACATGTGCTATTGAATT	
OriP #1	EBV	GTACTACCCGCATATAGATT	
OriP #2	EBV	GTACTACCCACATAGAGATT	x
EBNA1 #1	EBV	GGCCCTGATCCTGAGCCGCC	x
EBNA1 #2	EBV	GTGTGAATCATGTCTGACGA	x
US6 #1	HCMV	GTCACGCGAAGACCGCTCAC	
US6 #2	HCMV	GAAGACCGCTCACCGGGGGT	
US7 #1	HCMV	GATACCACGATGTTGGACTT	
US11 #1	HCMV	GCCCTCTGGGCCCCGGTCGC	
UL44 #1	HCMV	GGATCGCAAGACGCGTCTCT	
UL44 #2	HCMV	GCGGCTGAAGCCGTACAAGA	
UL44 #3	HCMV	GGATCACAGATCGCAGCTGC	
UL44 #4	HCMV	GCCGTCGCTTATCTTGCAAA	
UL54 #1	HCMV	GTCACGCCGCCGCTCAGATA	
UL54 #2	HCMV	GCCCGAGCCCTGCGCGGAGC	
UL54 #3	HCMV	GTCATGTTCGACGGTCAGAC	
UL54 #4	HCMV	GTGTCCTTGGCGCGAGACCC	
UL57 #1	HCMV	GGAACTAACCGCGCTAGCGC	
UL57 #2	HCMV	GGTGATCGCTCCTCTGTTGG	
UL57 #3	HCMV	GTGGCTTTTACCGAGGATCA	
UL57 #4	HCMV	GTTGGAGGCCTACACGCCCG	
UL70 #1	HCMV	GGGAAGACGCAGTGGTCCGT	
UL70 #2	HCMV	GTGCTCGGACTCGCGGCGGG	
UL70 #3	HCMV	GACTGGCGATGAGCGCCGAG	
UL70 #4	HCMV	GGTGCGCGAATTCAAGCACC	
UL84 #1	HCMV	GCGGGCCCGATTCCGAAGGT	
UL84 #2	HCMV	GCGTCGACAGAGCTCGGCGT	
UL84 #3	HCMV	GCGTCGACAGAGCTCGGCGT	
UL84 #4	HCMV	GACGCCCATCATGGTCGTGG	
UL86 #1	HCMV	GACGTGCGTCAGAAAGTCAG	
UL86 #2	HCMV	GGCCTCGAACATCTCCTCGC	
UL86 #3	HCMV	GAAGTGAATATTGTAACGCT	
UL86 #4	HCMV	GACCTCAATAAACTGACTAC	
UL105 #1	HCMV	GATCAAGGCGTCGTCGTACT	
UL105 #2	HCMV	GACGATCCGTTCGATCTTGG	
UL105 #3	HCMV	GACTGATGGAGCGAAACCAC	
UL105 #4	HCMV	GTGAACGGTCGTCGTTGCCG	
UL5 #1	HSV-1	GGGTTTCTGTCCGTCTAGC	
UL5 #2	HSV-1	gAAATTTTACGTCTATGCAC	
UL5 #3	HSV-1	GTCTAGCTGGCGCTCCCCGC	
UL5 #4	HSV-1	GGAACGGCTGGTCGGTCCC	
UL8 #1	HSV-1	GGACACCGCAGATATCGTGT	x

UL8 #2	HSV-1	gCCATTACCCTTTACGCGGT	
UL8 #3	HSV-1	GGGGCAGCCATACCGCGTAA	
UL8 #4	HSV-1	GGAAGTACTCGCGAGCGCG	
UL9 #1	HSV-1	gTCGCACTCGTCGTCCCCAA	
UL9 #2	HSV-1	GTCGAGCGTATCGCTAGCG	
UL9 #3	HSV-1	GCGGAGTCGGGAGATCCTC	
UL9 #4	HSV-1	gCTAGCGCGGATGTTGTACG	
UL15 #1	HSV-1	gACTTAAGGTGGGCGCGGAC	
UL15 #2	HSV-1	GCTCGCGCGTGTCCGTCCGA	
UL15 #3	HSV-1	gACCACGGTCTGGTGGCGCT	
UL15 #4	HSV-1	GCGGTCGCGAAATCTAAAAA	
UL27 #1	HSV-1	GGCGCCGGTGGTTCGTCGTA	
UL27 #2	HSV-1	GCGCCCATACGACGAACCAC	
UL27 #3	HSV-1	GTTCGTCGTATGGGCGCTCT	
UL27 #4	HSV-1	GGCGCTCTTGGGGTTGACGC	
UL29 #1	HSV-1	GCGAGCGTACACGTATCCC	x
UL29 #2	HSV-1	GCGAGCGTACACGTATCCCA	
UL29 #3	HSV-1	GCGTTACTGTCGGCACGCAG	
UL29 #4	HSV-1	GAGCGTACACGTATCCCAG	
UL30 #1	HSV-1	gTCCGGGTTTTTTGCGCCCG	
UL30 #2	HSV-1	gAAAAAACCCGGACGCCGCC	
UL30 #3	HSV-1	GCCGGCGGGCGCAAAAAACC	
UL30 #4	HSV-1	GCTTCTGTTGCGTCCCGACT	
UL36 #1	HSV-1	GCACTCCACGATGGAACGCG	
UL36 #2	HSV-1	GGGGGGGATCGCGACATCG	
UL36 #3	HSV-1	GAACCAGTTCGCGCCCGACC	
UL36 #4	HSV-1	GCACTCCACGATGGAACGCG	
UL37 #1	HSV-1	GCTGCATAGGTCCGTCCGA	
UL37 #2	HSV-1	GCCTATTGGCGAGCCTAGC	
UL37 #3	HSV-1	GGACCTATGCAGCGCCTAT	
UL42 #1	HSV-1	GACGCGTCGGACGCGTCCCT	
UL42 #2	HSV-1	gTCCCCCGTGGAGGACGCGT	
UL42 #3	HSV-1	gATTAAGTTCGGCGCCCTGC	
UL42 #4	HSV-1	gTGCAGGGCGCCGAACTTAA	
UL52 #1	HSV-1	gCCGTCGGTCGCATAAAGCG	x
UL52 #2	HSV-1	GAGGTAATAACGCACCCGT	
UL52 #3	HSV-1	gACCGCGCTTTATGCGACCG	
UL52 #4	HSV-1	gCCGCGCTTTATGCGACCGA	
UL54 #1	HSV-1	GTCCTCGTCCAGATCGCTGT	
UL54 #2	HSV-1	GTCCGATTCCAGGTCGTCG	
UL54 #3	HSV-1	gTTCCATGTCCTCGTCCGAC	
UL54 #4	HSV-1	GCGGCGTCGAGTATCGGCTC	
US3 #1	HSV-1	gTTTTAGAGCAGCCCCCGCG	
US3 #2	HSV-1	gTTGTAAGGCCACGCACGGC	
US3 #3	HSV-1	ATTTACGGGGGGGTAGGTCA[b]	
US3 #4	HSV-1	gATTTGTAAGGCCACGCACG	
US8 #1	HSV-1	GGGGATAGCAGCCGATCTT	
US8 #2	HSV-1	GGGAACGCCCAAAACGTCC	
US8 #3	HSV-1	gTCCCCGGGACATGGATCGC	
US8 #4	HSV-1	GCCCCGCGATCCATGTCCCG	

[a]lowercase g residue does not correlate to the genomic DNA sequence

[b] gRNA was mistakingly designed without a 'G' start residue
