# Supplementary material for: CRISPR/Cas9-Mediated Genome Editing of Herpesviruses Limits Productive and Latent Infections
Source: PLoS Pathog. 2016 Jun 30;12(6):e1005701. doi: 10.1371/journal.ppat.1005701 (PMC4928872; doi:10.1371/journal.ppat.1005701)
Supplement: S2 Table — (PDF) [file ppat.1005701.s006.pdf]

S2 Table. Primer sequences for off-target analysis

Off-target site	Off-target sequence[a]	# mismatches + location[b]	Chr.[c]	Position[d]	Fw primer (5'-3')	Rev Primer (5'-3')
EBV EBNA1 #1 OFF1	GCCTCTGGTCCTGAGCCGCCAGG	3MMs[2:4:8]	10	72646409	CCACCCCTAGCCAGTGATGG	TGCTTCCTTGCTTTCTCTGAGG
EBV EBNA1 #1 OFF2	TGCCCTCGTCCTGAGCCGCCGGG	3MMs[1:7:8]	18	47088444	GTCTCGGTTCCGGCGTCAG	CAATCCCGGCCGGTAAGACC
EBV EBNA1 #1 OFF3	TGCCTTAATCCTGAGCCGCCAAG	3MMs[1:5:7]	1	74674620	AGCCAAAATCAAAACTGCCTTC	TTCCTCAACCTCAATAGAAATAGGC
EBV EBNA1 #2 OFF1	GTTTGAGCCATGTCTGACGAGGG	3MMs[3:7:8]	X	16964258	GGAGGAATGAAGGTGAACATGGC	GAGCGGGGAAGGTTGTGTTG
EBV EBNA1 #2 OFF2	GTGAGAAGCATGTCTGACAAGAG	3MMs[4:8:19]	13	109050456	GGGCTTAGTGGGCTGTGTGG	TCTGACCCTATCCTGGACCTAAC
EBV EBNA1 #2 OFF3	CTGTGAAACATGTCTGAGGATGG	3MMs[1:8:18]	19	56630147	CCAGGTTCCTGCAAAGTGTG	GGTGCAGGGGAGGTATTGTG
EBV OriP #2 OFF1	GTATTACTCACATAGAAATTTGG	3MMs[4:8:17]	5	85504537	GATGGAGAAAACAGGGTCAGTTC	ATAGGCATGCACCACCACAC
EBV OriP #2 OFF2	GTACCACCCTCATAGAGAGTTAG	3MMs[5:10:19]	X	66266042	GGTGACTGATCCCATGAAGCC	CCTCAGCTGGGTGGTAGGAC
EBV OriP #2 OFF3	GAAAGGCCCACATAGAGATTTAG	4MMs[2:4:5:6]	7	114977697	TAATTTGGGGGAGGGAGGTG	AAGAAAACCCTTGACAAGTCAGC
HCMV UL44 #2 OFF1	GCCGATGAAGGCGTACAAGAAAG	3MMs[3:5:11]	17	38711140	AGTCCAGGCAGAAGAGTCGC	AGCGCAACAAGGCCATCAAG
HCMV UL44 #2 OFF2	GAGGCTGGGGCCGTACAAGGCGG	4MMs[2:8:9:20]	2	239755863	CCCCAACCACTTCAAACGCG	GACGCCCTTCCCGAACCTTC
HCMV UL44 #2 OFF3	GGGGCTTCAGCCTTACAAGAGAG	4MMs[2:7:8:13]	16	71859806	GGACAGAGCAACAAAGGGCTG	CGTGGTGGTGCATGCCTG
HCMV UL57 #3 OFF1	GTAGCTTCTACCTAGGATCACAG	3MMs[3:8:13]	6	83883840	TTCACCCTGTTAGCCAGGAG	CAGAACAGAGTGTGGGTGCATAC
HCMV UL57 #3 OFF2	GTGGGTTTCACTGAGGATCACAG	3MMs[5:9:12]	2	23532359	GAACCCGACCTCTGTGAGCC	GAAGGAGCGAGAGTGAGGCC
HCMV UL57 #3 OFF3	ATGCCTTATACAGAGGATCATAG	4MMs[1:4:8:12]	12	30161448	GCCCAGGGAAGAACAAATGGC	ACCAAAAGAGGCTGCAGTTCAC
HCMV UL105 #3 OFF1	GGCTGAGGGAGGGAAACCACAGG	3MMs[2:7:12]	9	139337041	AGTGTGTGAGGCTGTGAGGG	AGCTGCCTTTCTCCTCCGG
HCMV UL105 #3 OFF2	GAATGATGCAGGGAAACCACTAG	3MMs[3:9:12]	2	116463167	ACGTTACTGCTGGTGCCAAG	CCCTGCAACTAAAAGCAGGAAC
HCMV UL105 #3 OFF3	TACTGATGGATGGAAACCACAGG	3MMs[1:11:12]	5	158105421	CCCTCATCGCCAAAGGAAGG	CCCACATCCCTGGTAGCAAAC
HSV-1 UL52 #1 OFF1	CCCCCCGGTAGCATAAAGGGGAG	4MMs [4:5:10:19]	14	100012444	CAACTCCCAGCCTCCAACTC	CGCCTCCCCACTGTTCTTAC
HSV-1 UL52 #1 OFF2	CAGGGCTGTATGCGTCCGACGGG	4MMs [2:4:8:15]	8	145202603	TCTTATCGCGTCCCGAGTTC	TCATTTGCAAAGCAGCAGTG
HSV-1 UL52 #1 OFF3	TCGGTCGCTCGCAGAAAGCGGGG	4MMs [1:3:8:14]	6	161413133	GCCAGGCTGCAGCTTACTG	AGTCGCCGGACAGACTCAG
HSV-1 UL8 #1 OFF1	GAAAACCTCAGATATCATGTCAG	4MMs [2:4:8:17]	5	75853084	CCAAGCCGTGGAACTCTACC	TTTGCCAAAATCTGGACTTGATAC
HSV-1 UL8 #1 OFF2	GAAGACGGCAGATATCCTGTGAG	4MMs [2:4:7:17]	8	17324699	CAAATGCCTAAGGATTGTCTTGC	GAACCGGGTAGGCAGAGG
HSV-1 UL8 #1 OFF3	GGAAATCACAGATATCGTGCTGG	4MMs [4:6:8:20]	15	31604828	ACTGGAGAGGGCCGTATGTG	TCTAGGCCCTCTTCCTTCACC
HSV-1 UL29 #1 OFF1	GGTGAGCGTCCACGTGTCCCCAG	3MMs [3:10:16]	6	31995010	TGCGGCCTGTCCTCTATAAC	CCACAGGGAATTCGAAGGAC
HSV-1 UL29 #1 OFF2	GGTGAGCCTACACGGAACCCTGG	4MMs [3:8:15:17]	2	202840343	ACCCACGGGCAAATACTCTC	ACGCCACCTCCTCAGAAAAC
HSV-1 UL29 #1 OFF3	GACGAGCTTACAGGTTTCCCAAG	4MMs [2:8:13:16]	6	45524414	GCTTTGTAAACCCCTGTTTGG	TGGACTCTGACAGGGGGAAG

[a]potential off-target sequence. Mismatches as compared to the gRNA are indicated in bold and red whereas the PAM sequence is indicated in bold

[b]number of mismatches (MM) and location of mismatch as compared to the gRNA is indicated

[c]chromosome

[d]nucleotide location on the chromosome from the previous column
