# Supplementary material for: CRISPR/Cas9-Mediated Genome Editing of Herpesviruses Limits Productive and Latent Infections
Source: PLoS Pathog. 2016 Jun 30;12(6):e1005701. doi: 10.1371/journal.ppat.1005701 (PMC4928872; doi:10.1371/journal.ppat.1005701)
Supplement: S3 Table — (PDF) [file ppat.1005701.s007.pdf]

S3 Table. No signs for off-targeting activity of anti-HCMV and anti-EBV gRNAs

			gRNA-positive cells			gRNA-negative cells			
**Off-target site**	**Off-target sequence[a]**	**# mismatches + location[b]**	**# sequences total[c]**	**# mutated[d]**	**% mutated**	**# sequences total[c]**	**# mutated[d]**	**% mutated**	**gRNA-pos minus gRNA-neg[e]**
EBNA1 #1 OFF1	G**C**C**T**CTG**G**TCCTGAGCCGCC**AGG**	3MMs[2:4:8]	1930	5	0,26	1024	3	0,29	-0,03
EBNA1 #1 OFF2	**T**GCCCT**CG**TCCTGAGCCGCC**GGG**	3MMs[1:7:8]	2558	6	0,23	1300	5	0,38	-0,15
EBNA1 #1 OFF3	**T**GCC**T**T**A**ATCCTGAGCCGCC**AAG**	3MMs[1:5:7]	1665	14	0,84	1110	10	0,90	-0,06
EBNA1 #2 OFF1	GT**T**TGA**GC**CATGTCTGACGA**GGG**	3MMs[3:7:8]	1628	52	3,19	904	33	3,65	-0,46
EBNA1 #2 OFF2	GTG**A**GAA**G**CATGTCTGAC**A**A**GAG**	3MMs[4:8:19]	1755	20	1,14	953	17	1,78	-0,64
EBNA1 #2 OFF3	**C**TGTGAA**A**CATGTCTGA**G**GA**TGG**	3MMs[1:8:18]	2449	13	0,53	1182	12	1,02	-0,48
OriP #2 OFF1	GTA**T**TAC**T**CACATAGA**A**ATT**TGG**	3MMs[4:8:17]	2085	13	0,62	1089	11	1,01	-0,39
OriP #2 OFF2	GTAC**C**ACCC**T**CATAGAGA**G**T**TAG**	3MMs[5:10:19]	2414	19	0,79	1133	13	1,15	-0,36
OriP #2 OFF3	G**A**A**AGG**CCCACATAGAGATT**TAG**	4MMs[2:4:5:6]	1749	10	0,57	937	5	0,53	0,04
UL44 #2 OFF1	GC**C**G**A**TGAAG**G**CGTACAAGA**AAG**	3MMs[3:5:11]	2348	20	0,85	1186	8	0,67	0,18
UL44 #2 OFF2	G**A**GGCTG**GG**GCCGTACAAG**GCGG**	4MMs[2:8:9:20]	603	2	0,33	353	2	0,57	-0,23
UL44 #2 OFF3	G**G**GGCT**TC**AGCC**T**TACAAGA**GAG**	4MMs[2:7:8:13]	1229	10	0,81	617	2	0,32	0,49
UL57 #3 OFF1	GT**A**GCTT**C**TACC**T**AGGATCA**CAG**	3MMs[3:8:13]	2132	11	0,52	1009	7	0,69	-0,18
UL57 #3 OFF2	GTGG**G**TTT**C**AC**T**GAGGATCA**CAG**	3MMs[5:9:12]	2303	11	0,48	1158	10	0,86	-0,39
UL57 #3 OFF3	**A**TG**C**CTT**A**TAC**A**GAGGATCA**TAG**	4MMs[1:4:8:12]	2326	16	0,69	1231	11	0,89	-0,21
UL105 #3 OFF1	G**G**CTGA**G**GGAG**G**GAAACCAC**AGG**	3MMs[2:7:12]	597	4	0,67	438	6	1,37	-0,70
UL105 #3 OFF2	GA**A**TGATG**C**AG**G**GAAACCAC**TAG**	3MMs[3:9:12]	2205	17	0,77	1027	8	0,78	-0,01
UL105 #3 OFF3	**T**ACTGATGGA**TG**GAAACCAC**AGG**	3MMs[1:11:12]	1451	11	0,76	668	8	1,20	-0,44

[a]potential off-target sequence. Mismatches as compared to the gRNA are indicated in bold and red whereas the PAM sequence is indicated in bold

[b]number of mismatches (MM) and location of mismatch as compared to the gRNA is indicated

[c]Number of sequences analyzed

[d]Number of sequences displaying alterations as compared to the reference sequence. A sequence scored as 'mutated' when there was a mismatch/indel 2bp up and downstream of the Cas9 cleavage site

[e]Percentage of mismatches that could have occurred by CRISPR/Cas9 mediated gene editing. The percentage of edited sequences in gRNA-expressing cells was subtracted from the percentage of gRNA-negative cells. A negative value denotes a higher percentage of mutations in gRNA-negative cells as compared to gRNA-expressing cells.
